# Perceptual resolution of ambiguity: A divisive normalization account for both interocular color grouping and difference enhancement

**DOI:** 10.1167/jov.26.1.8

**Published:** 2026-01-13

**Authors:** Jaelyn R. Peiso, Stephanie E. Palmer, Steven K. Shevell

**Affiliations:** 1 Department of Psychology, Institute for Mind & Biology, University of Chicago, Chicago, IL, USA; 2 Department of Organismal Biology & Anatomy, Department of Physics, Physics Frontier Center for Living Systems, University of Chicago, Chicago, IL, USA; 3 Department of Psychology, Department of Ophthalmology & Visual Science, Institute for Mind & Biology, University of Chicago, Chicago, IL, USA

**Keywords:** binocular rivalry, divisive normalization, ambiguity resolution, interocular-switch rivalry

## Abstract

Our visual system usually provides a unique and functional representation of the external world. At times, however, there is more than one compelling interpretation of the same retinal stimulus; in this case, neural populations compete for perceptual dominance to resolve ambiguity. Spatial and temporal context can guide this perceptual experience. Recent evidence shows that ambiguous retinal stimuli are sometimes resolved by enhancing either similarities or differences among multiple ambiguous stimuli. Although rivalry has traditionally been attributed to differences in stimulus strength, color vision introduces nonlinearities that are difficult to reconcile with luminance-based models. Here, it is shown that a tuned, divisive normalization framework can explain how perceptual selection can flexibly yield either similarity-based “grouped” percepts or difference-enhanced percepts during binocular rivalry. Empirical and simulated results show that divisive normalization can account for perceptual representations of either similarity enhancement (so-called grouping) or difference enhancement, offering a unified framework for opposite perceptual outcomes.

## Introduction

The human visual system continuously interprets a stream of ambiguous sensory input to form coherent perceptual experiences (Brascamp & Shevell, 2021). When faced with conflicting inputs—such as incompatible images presented to each eye—perception frequently alternates between the two inputs rather than blending them. For example, observers who are shown isoluminant red and green patches frequently report red or green percepts that alternate over time rather than a stable yellow percept. This phenomenon, known as binocular rivalry, offers a unique lens into how the brain resolves competition between neural populations encoding incompatible stimuli. A longstanding framework posits that perceptual selection of the dominant percept hinges on stimulus strength (e.g., luminance intensity or contrast), with stronger inputs suppressing weaker ones (Brascamp, Klink, & Levelt, 2015; Levelt, 1966). However, this account faces a critical limitation for understanding perceptual selection in the context of chromatic rivalry because color perception is inherently nonlinear and cannot be reduced to linear, luminance-like strength metrics (Bujack, Teti, Miller, Caffrey, & Turton, 2022). How is stimulus strength determined for different hues of the same luminance?

Chromatic signals are encoded by cone-opponent mechanisms and are shaped by context (e.g., adaptation and surround effects; Werner, 2014), so “strength” cannot be defined by intensity or contrast like it can for luminance. Color spaces are non-Riemannian—the “distance” between hues depends on both their magnitudes and their relative angles (Bujack et al., 2022), in addition to contextual factors that further shape the underlying neural responses. These nonlinearities undermine any single scalar “strength” for isoluminant hues. To overcome this, the “strength” of the competing chromatic signals is estimated via divisive normalization. Here, responses from similarly tuned neurons are pooled across the current stimulus, enabling contextual reweighting. In this view, a hue's signal is “strong” to the extent that it is less attenuated by its normalization pool in the present scene—an account that naturally captures both similarity-based grouping and difference-enhanced percepts during interocular-switch rivalry (ISR).

Understanding perceptual selection during the resolution of competing chromatic signals calls for a binocular rivalry model that goes beyond simple strength‐based accounts. Traditionally studied with luminance stimuli, binocular rivalry exhibits systematic, nonrandom alternations between percepts. Early theories explained this phenomenon solely through interocular suppression mediated by reciprocal inhibition between monocular neurons (Blake, 1989; Lehky, 1988). However, observations of coherent percepts with patchwork stimuli and extended dominance durations during ISR challenge this simple account. Perception of coherent percepts persists with patchwork stimuli, which require selective binocular integration (Kovács, Papathomas, Yang, & Fehér, 1996). In experiments using ISR, where stimuli rapidly alternate between eyes, dominance durations extend much longer than the swapping rate (Christiansen, D'Antona, & Shevell, 2017; Logothetis, Leopold, & Sheinberg, 1996). These results suggest a more complex network of rivalry mechanisms that extend beyond early visual areas to include extrastriate regions such as V4 (Blake & Logothetis, 2002; Desimone, 1998; Duncan, 2006; Freeman, 2005; Kastner & Ungerleider, 2001; Kim, Hong, Shevell, & Shim, 2020; Tong, Meng, & Blake, 2006; Wilson, 2003). Interocular grouping (IOG) can be characterized as a perceptual bias: When faced with multiple rivalrous regions, the brain preferentially binds them into a single, identical percept (Kovács et al., 1996). Although IOG can occur with both luminance and chromatic stimuli, chromatically defined stimuli more robustly elicit IOG than luminance-defined features, such as motion (Papathomas, Kovács, & Conway, 2004), suggesting that distinct computations may underlie perceptual resolution in chromatic rivalry. Additionally, most IOG experiments in chromatic rivalry have measured only grouped, similarity-enhanced percepts in which rivalrous regions synchronously resolve to share identical perceptual properties (e.g., Kovács et al., 1996; Lee & Blake, 2004; Slezak & Shevell, 2018). Difference-enhanced percepts had not been measured during IOG experiments until recent work found that they could dominate perception, while the occurrence of grouped, similarity-enhanced percepts was diminished (Peiso & Shevell, 2020). Together, these findings make chromatic IOG a powerful paradigm for probing how contextual factors steer perceptual selection using binocular rivalry.

**Figure 1.**
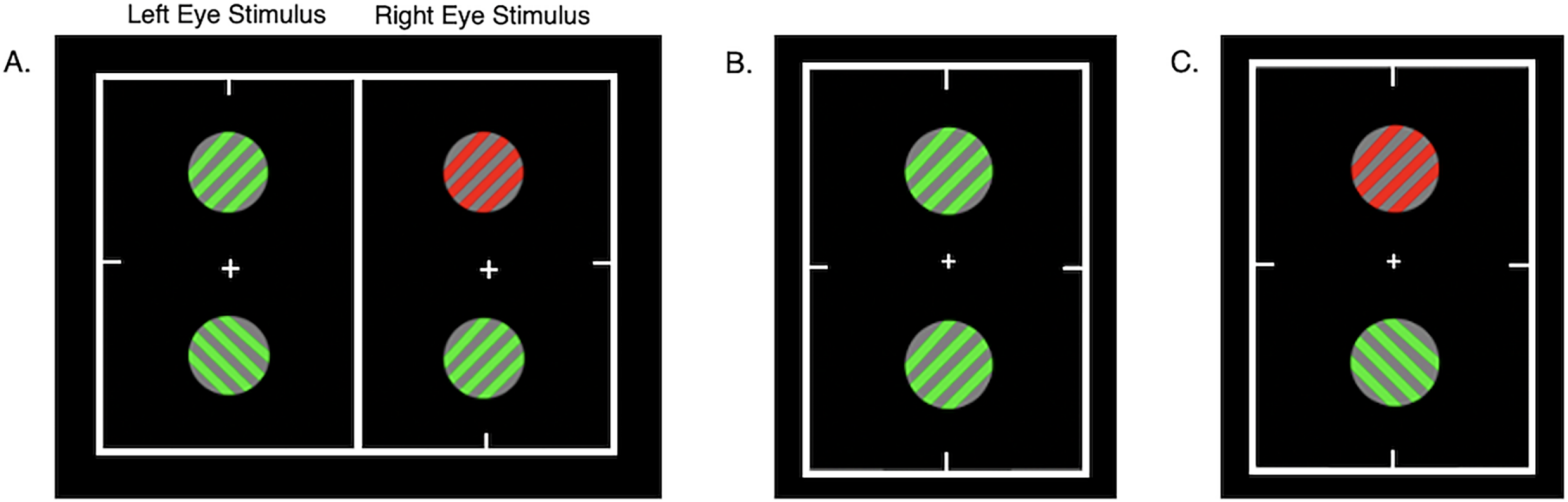
Example stimulus and measured similarity-enhanced and difference-enhanced percepts. (**A**) Rivalrous chromatic grating stimuli presented dichoptically inside fusion boxes with Nonius lines. (**B**) A similarity-enhanced fused percept. (**C**) A difference-enhanced fused percept (Peiso & Shevell, 2020).

In the study revealing difference enhancement, participants viewed two equiluminant gratings in rivalry—one positioned above and one below fixation (Figure 1A). This design allowed for two potential perceptual outcomes: a similarity-enhanced grouped percept (Figure 1B) or a difference-enhanced percept (Figure 1C). Rivalry was induced using either different colors (for the gratings above fixation) or orthogonal orientations (for those below fixation). Notably, the stimulus was constructed so that the rivalrous gratings could resolve as either identical in color and orientation (Figure 1B) or different in both aspects (Figure 1C). Contrary to expectations based on a bias toward perceptual similarity, difference-enhanced percepts (Figure 1C) were seen significantly more often than similarity-enhanced percepts (Figure 1B). This finding raises the question of whether IOG is fundamentally biased toward enhancing similarity or instead reflects a more flexible process that can also emphasize differences. Because perceptual selection can depend on coherent object-level representations rather than eye of origin alone, such flexibility may reflect higher-level mechanisms sensitive to contextual structure (Buckthought, Kirsch, Fesi, & Mendola, 2021; Mitchell, Stoner, & Reynolds, 2004). Divisive normalization offers a plausible framework for the unexpected dominance of difference-enhanced percepts.

Divisive normalization offers a plausible framework for the unexpected dominance of difference-enhanced percepts. Developed initially to account for nonlinear response properties of V1 neurons (e.g., luminance contrast gain control and cross-orientation suppression), divisive normalization models a neuron's activity as its input divided by the pooled activity of neighboring neurons plus a constant (Heeger, 1992; Schwartz & Simoncelli, 2001). This fundamental computational motif extends across brain regions and sensory modalities, mediating neural competition by biasing responses in favor of task-relevant signals (Lee & Maunsell, 2009; Louie & Glimcher, 2019; Reynolds & Heeger, 2009). More recent models suggest that divisive normalization pools are not static and instead dynamically adapt based on task demands and contextual dependencies, allowing for flexible reweighting of inputs to optimize perceptual processing (Aqil, Knapen, & Dumoulin, 2021; Beuth & Hamker, 2015; Coen-Cagli, Dayan, & Schwartz, 2012; Coen-Cagli, Kohn, & Schwartz, 2015; Louie & Glimcher, 2019; Northoff & Mushiake, 2020; Schwartz & Coen-Cagli, 2013). Originally characterized at the cellular level, divisive normalization has since been extended to population dynamics (Aqil, Knapen, & Dumoulin, 2021; Carandini & Heeger, 2012; Louie, Khaw, & Glimcher, 2013; Reddy, Kanwisher, & VanRullen, 2009), where it explains nonlinearities across neuronal populations, regulates neural competition in favor of task-relevant signals (Reynolds & Heeger, 2009; White, Rolfs, & Carrasco, 2015), and enables context to modulate neural responses (Louie & Glimcher, 2019). Furthermore, feature-tuned divisive normalization provides a framework for understanding how perceptual selection emerges due to biased competition (Aqil, Knapen, & Dumoulin, 2021; Li, 1999). Here, neural responses are divisively scaled by the pooled activity of similarly tuned neurons across the attended visual field. These neural pools provide a mechanism for perceptual biases, such as IOG, that aid in the perceptual resolution of neural ambiguity. The chromatic IOG model presented here is grounded in this theoretically parsimonious framework.

### Overview of the computational model

This framework integrates divisive normalization, competition, attention, adaptation, and recovery to address the neural mechanisms underlying perceptual selection during rivalry. Models of rivalry typically include additional computational factors, such as adaptation, intrinsic noise, and attention, even when these are not directly manipulated in a given experiment, because they are necessary to produce realistic dynamics (Brascamp & Blake, 2012; Brascamp, Van Ee, Noest, Jacobs, & van den Berg, 2006; Shpiro, Moreno‑Bote, Rubin, & Rinzel, 2009; Wilson, 2003; Zhang, Jamison, Engel, He, & He, 2011). Here, feature-tuned, pooled divisive normalization models each neuron's output as divisively normalized by the pooled activity of similarly tuned neurons and itself, providing a flexible mechanism for contextual reweighting. It has been shown that binocular rivalry phenomena are attention-dependent: When attention is diverted away, perceptual alternations largely cease, and perception collapses into a stable mixture (Brascamp & Blake, 2012; Zhang et al., 2011). Drawing on evidence that attention enhances positive serial dependence in vision to bias perception toward recent experiences (Fischer & Whitney, 2014; Manassi & Whitney, 2024), the model here implements gain probabilistically with a slight advantage for the currently dominant percept. Consistent with models of rivalry adaptation (Shpiro et al., 2009; Wilson 2003), the dominant (attended) representation adapts strongly, whereas the suppressed channel recovers (asymmetric dynamics), promoting alternations without gridlock or instability. This reinforces temporal continuity and preserves sensitivity under ambiguous sensory conditions, reducing the risks of perceptual gridlock or unstable fluctuations. The present framework is novel in using pooled divisive normalization to explicitly explain chromatic interocular grouping during binocular rivalry, an aspect not previously accounted for by canonical normalization models. A step-by-step walkthrough of the model is below, detailing its mathematical underpinnings and linking each component to empirical evidence that motivates these choices.

#### Reweighting sensory signals with a normalization process

The first step involves divisive normalization (Carandini & Heeger, 2012; Reynolds & Heeger, 2009). By pooling similarly tuned neurons, divisive normalization enables contextual reweighting (Aqil, Knapen, & Dumoulin, 2021), which is critical for resolving perceptual ambiguities during binocular rivalry. Sensory signals corresponding to different colors—say, red (*S_R_*) and green (*S_G_*)—are normalized to produce the outputs (*Ŝ_R_* and *Ŝ_G_*), which are the signals conveying red and green, respectively. To focus processing on regions where rivalry occurs, these signals are convolved with a spatial filter, *f*($\bar{x}$), that isolates the rivalrous regions from the rest of the attended visual field ($\bar{x}$ = [*x₁*, *x₂*]). Formally, this is

(1) $$\begin{array}{c} {\hat{S}}_{R}=\frac{\sum_{\bar{x}}f\left(\bar{x}\right)\circ S_{R}\left(\bar{x}\right)}{\sum_{\bar{x}}S_{R}\left(\bar{x}\right)}+\eta_{R},\; \end{array}$$

(2) $$\begin{array}{c} {\hat{S}}_{G}=\frac{\sum_{\bar{x}}f\left(\bar{x}\right)\circ S_{G}\left(\bar{x}\right)}{\sum_{\bar{x}}S_{G}\left(\bar{x}\right)}+\eta_{G}.\; \end{array}$$

This operation integrates local sensory information over space as dictated by *f*($\bar{x}$), effectively enhancing regions with competing inputs while attenuating homogeneous areas—much like an attentional mechanism (Reynolds & Heeger, 2009). Related early‑vision work on preattentive segmentation/correspondence likewise supports an early selection stage (Zhaoping, 2002). The spatial weighting *f*($\bar{x}$) here is used as a saliency-inspired gate: Motivated by V1 computations in Li's model, it assigns higher weight to locations with strong local feature contrast—locations that, in our displays, coincide with the rivalrous patches. In this sense, *f*($\bar{x}$) can be viewed as an early, feed-forward selection signal that guides subsequent normalization at higher stages (Li, 1999, Li, 2002).

The numerator is obtained by summing the masked red or green signal over both eyes (i.e., binocular pooling across eyes) and represents the chromatic signals in the rivalrous locations. The denominator sums the total chromatic signal across the entire visual stimulus in both eyes. Dividing the two yields a normalized signal that reflects each color's relative weight within rivalrous regions, after collapsing across eye of origin, scaled by its overall value across the full stimulus. This operation is deterministic and fully defined by stimulus chromaticities and the spatial mask. In this way, divisive normalization acts as an early contextual reweighting mechanism.

Next, parameter η_C_ represents intrinsic sensory noise that varies with signal strength and perceptual signal (*S_C_*) to account for real-world neural variability and is given by

(3) $$\begin{array}{c} \eta_{c}=S_{C}\cdot \xi_{c},\xi ∼N{\left(0,\sigma^{2}\right)}_{\left[a,b\right]}.\; \end{array}$$

As shown in Equation 3, noise (η_C_) depends both on the current signal, *S*_C_, and a random variable, ξ_C_, drawn from a truncated normal distribution. Bounding parameters, [*a*, *b*], ensure that perturbations remain within a biologically plausible range, preventing unrealistic dynamics such as negative neural activity or excessively high noise levels. Intrinsic neural variability contributes to perceptual variability in binocular rivalry (e.g., Baker & Richard, 2019; Brascamp et al., 2006; Kim, Grabowecky, & Suzuki, 2006), motivating the inclusion of internal noise in our model. Physiologically, trial-to-trial variability is substantial and is quenched at stimulus onset (Churchland et al., 2010). Psychophysically, chromatic noise-masking patterns are consistent with mechanism-selective processing in color pathways, and equivalent-noise methods provide a principled way to infer internal noise (Gegenfurtner & Kiper, 1992; Lu & Dosher, 1999; Sankeralli & Mullen, 1997). Accordingly, an additive, signal-dependent noise (η_C_) is injected every Δ*t*; η_C_ is resampled from *N*(0, σ^2^)_[*a*,*b*]_ and added to the channel activity.

#### Multiplicative gain and neural competition

The second component of the framework captures how multiplicative gain and winner-take-all (WTA) neural competition interact to govern rivalry dynamics between normalized sensory representations. In this model, stronger sensory signals dominate perceptual awareness—a principle that aligns with theories of biased competition and attentional selection (Desimone & Duncan, 1995; Heeger, 1992). Stochastic fluctuations and dynamic gain modulation allow the system to switch between percepts, reflecting the inherent variability observed in neural activity (White et al., 2015; Wilson, 2003).

After normalization, the system selects the stronger neural representation, that is, the maximum of the normalized signals (*Ŝ_R_* or *Ŝ_G_*) as the dominant percept (*P_D_*). To capture the influence of multiplicative gain, a gain multiplier is then applied probabilistically to one of the representations (*P_D_* or *P_S_*). Formalizing this process by amplifying the strength of the selected signal with a slight bias toward the already dominant percept reinforces its competitive advantage, giving

(4) $$\begin{array}{c} P_{D}\;or\;P_{S}=g\left(P_{D\;}or\;P_{S}\right).\; \end{array}$$

Next is a recursive comparison between the currently dominant (*P_D_*) and suppressed (*P_S_*) representations at each time step (Δ*t*):

(5) $$\begin{array}{ccc} & & \;P_{D}\left(t+\Delta t\right)=max\left(P_{D}\left(t\right)+\eta_{D}\left(t\right),P_{s}\left(t\right)+\eta_{S}\left(t\right)\right).\;\;\; \end{array}$$

Δ*t* captures the interval between sampling by the spatial-temporal integration process, selected based on empirical evidence regarding the temporal sampling rate of spatial attention (Davidson, Alais, van Boxtel, & Tsuchiya, 2018; Re, Inbar, Richter, & Landau, 2019). The use of a maxing function aligns closely with canonical neural circuits capable of performing diverse nonlinear computations, such as divisive normalization, Gaussian-like tuning, and max-like selection (Kouh & Poggio, 2008). The intrinsic noise components (η*_D_* and η*_S_*) independently add random fluctuations for the dominant and suppressed representations at each time step. This stochasticity captures the inherent variability in neural systems that is critical in driving perceptual switches during rivalry (Brascamp et al., 2015). The noise term is stochastic and modeled as the product of a random variable and the current signal (see Equation 3).

#### Adaptation, recovery, and intrinsic neural noise

The third component of the framework concerns the selective adaptation of the dominant neural representation and recovery of the previously suppressed representation. Adaptation mechanisms prevent prolonged dominance, ensuring dynamic flexibility in rivalry resolution and avoiding deterministic perceptual states for ambiguous stimuli (Kohn, 2007). Noisy neuronal adaptation is a key driver of the stochastic fluctuations in attention that underlie perceptual alternations in bistable stimuli (Dieter, Melnick, & Tadin, 2015; Shpiro et al., 2009; van Ee, 2009). As the dominant percept adapts, the suppressed percept recovers its strength and is continuously modulated by intrinsic noise, thereby maintaining dynamic competition (Brascamp et al., 2015).

Modeling the adaptation of the dominant percept (*P_D_*) as an exponential decay over time, governed by the time constant τ*_D_*, gives

(6) $$\begin{array}{c} \frac{dP_{D}\left(t\right)}{dt}=\frac{-P_{D}\left(t\right)}{\tau_{D}}+\eta_{D}\left(t\right).\; \end{array}$$

This adaptation process, represented by τ*_D_*, weakens the dominant representation gradually, preventing it from permanently suppressing the competing percept. Such decay is critical for ensuring the possibility of perceptual switches and aligns with empirical evidence of neural adaptation in the visual cortex (Kohn, 2007). To characterize the recovery process, describing a gradual return of the suppressed representation (*P_S_*) to its maximal strength (*P_M_*) gives

(7) $$\begin{array}{c} \frac{dP_{R}\left(t\right)}{dt}=\frac{P_{M}-P_{R}\left(t\right)}{\tau_{R}}+\eta_{R}\left(t\right),\; \end{array}$$

where intrinsic noise (η*_D_* and η*_R_*) introduces continuous stochastic fluctuations affecting both representations. This recovery reflects the rebalancing of competitive signals, consistent with models of perceptual bistability and rivalry resolution (Freeman, 2005; Tong et al., 2006). The recovery process is controlled by the time constant, τ*_R_*, which dictates the rate at which the suppressed representation regains strength. Significantly, τ*_R_* is independent of the adaptation time constant (τ*_D_*) for the dominant percept, allowing for asymmetries in the dynamics of decay and recovery. This distinction provides a biologically plausible mechanism for the observed variability in perceptual switches, where recovery may occur at a different pace than adaptation (Wilson, 2003).

This framework integrates divisive normalization, adaptation, noise, and recovery into a unified account of perceptual competition during binocular rivalry. By pooling similarly tuned neurons and reweighting their activity, the model can naturally account for both similarity-based interocular grouping and difference-enhanced percepts. WTA competition promotes single-percept dominance, whereas adaptation and recovery introduce the flexibility required for switching. Finally, intrinsic noise adds an element of unpredictability, preventing strictly deterministic dominance.

The experiments presented here leverage two complementary design choices to test the framework's predictions: ISR and patchwork stimuli. ISR and patchwork stimuli require pooling information from multiple spatially distributed and temporally swapped signals while disrupting eye-based rivalry. The implementation of these methodologies focuses the experimental investigation below on how contextual and feature-based factors modulate competition. Critically, the stimuli were designed to isolate contextual modulation via divisive normalization by manipulating the size of the normalization pools while keeping chromatic contrast at the borders between the background and disks constant. Moreover, the framework has been implemented as a simulation, and its results are presented alongside the empirical findings from Experiment 1, providing a critical evaluation of the theoretical framework.

## General methods

### Apparatus

Stimuli were displayed on a calibrated NEC MultiSync FP2141SB cathode ray tube (CRT) monitor driven by an iMac computer. Observers viewed the CRT through an eight-mirror haploscope, which presented different stimuli to corresponding retinotopic regions in each eye. A chin rest maintained an approximately 115-cm-long light path through the haploscope. To ensure stable fusion of the two images and to account for individual differences in interocular distance, observers adjusted the position of the final mirror set. Two Nonius lines facilitated image fusion, with the left eye presented with top and left Nonius lines and the right eye presented with bottom and right Nonius lines. A properly fused image exhibited one fixation point, horizontally aligned left and right Nonius lines, vertically aligned top and bottom Nonius lines, and a binocularly fused square frame.

### Observers

Five observers (three female, ages 23–32) provided written informed consent prior to participation, as required by the University of Chicago's Institutional Review Board. Observers were screened for normal stereoscopic vision using the Titmus Stereo Test and for normal color vision employing Ishihara plates and Rayleigh matches made with a Neitz anomaloscope. Data for both experiments were collected concurrently. All observers participated in both experiments and were naive to the experimental hypotheses.

### Stimuli

Stimuli were initially defined in MacLeod–Boynton color space based on spectroradiometric calibration to a standard photopic observer (Smith & Pokorny, 1975). Individual isoluminance was then empirically refined for each participant using repeated trials of heterochromatic flicker photometry (HFP). HFP is a method used to measure the spectral sensitivity of the human eye and define the human photopic luminosity function (Bone & Landrum, 2004; Lee, Martin, & Valberg, 1988; Wyszecki & Stiles, 1982). The HFP stimulus involved a single region (i.e., disk) oscillating at approximately 15 Hz between two distinct chromaticities. Observers adjusted the level of one light to minimize the perception of flicker.

Each observer completed five HFP repetitions for three chromaticity pairs: red-appearing/green-appearing (R/G), blue-appearing/green-appearing (B/G), and blue-appearing/red-appearing (B/R) on 3 separate days. The five measurements per color pair were averaged, resulting in three daily means. After taking the average of these daily means, a final analytical check was made using the measured R/G and B/G equiluminant ratios to calculate a predicted B/R ratio. This calculated B/R ratio was compared to the measured B/R ratio, allowing for a deviation of ± 10%.

**Figure 2.**
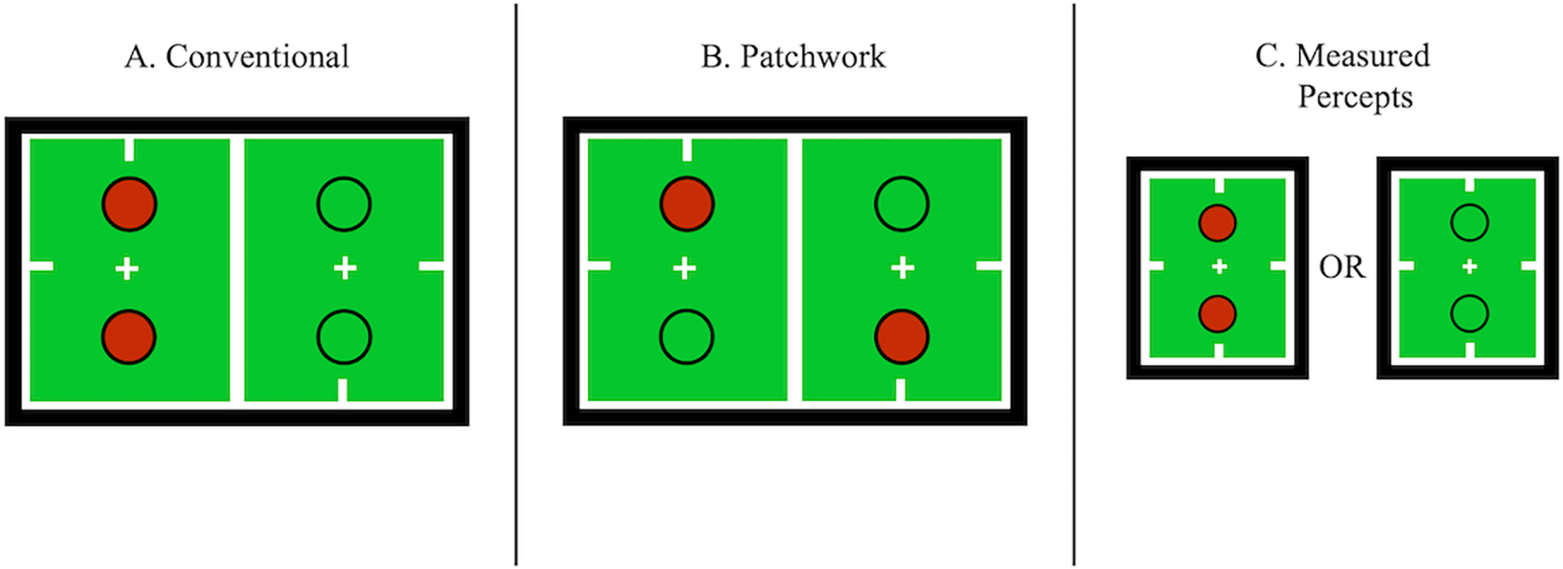
Conventional and patchwork presentations. (**A**) Conventional presentation refers to each eye receiving an identical stimulus in both rivalrous regions. (**B**) Patchwork presentation refers to each eye receiving a different stimulus in each rivalrous region. (**C**) Measured percepts from stimulus **A** or **B**.

All stimuli were generated in MATLAB as indexed images for efficient rendering. Chromatically defined stimuli were presented in ISR, which entails swapping stimuli between the two eyes at a rate of 3.75 Hz (cycles/second; two eye swaps per cycle), or 7.5 swaps per second (Christiansen et al., 2017). ISR was used in the experiments to eliminate differential adaptation between the two eyes. Differential adaptation can occur when each eye is presented with a static stimulus for an extended period, leading to varying levels of neural adaptation that can bias perceptual dominance (Blake & Overton, 1979; Christiansen et al., 2017; Logothetis et al., 1996). Only patchwork stimuli requiring binocular integration were tested because previous studies consistently found no significant differences in dominance times between conventional (Figure 2A) and patchwork (Figure 2B) stimulus configurations when presented in ISR (Lange & Shevell, 2020; Lee, Slezak, & Shevell, 2022; Peiso & Shevell, 2020; Slezak & Shevell, 2018; Shevell, 2019; Zhang, Slezak, Wang, & Shevell, 2021).

**Figure 3.**
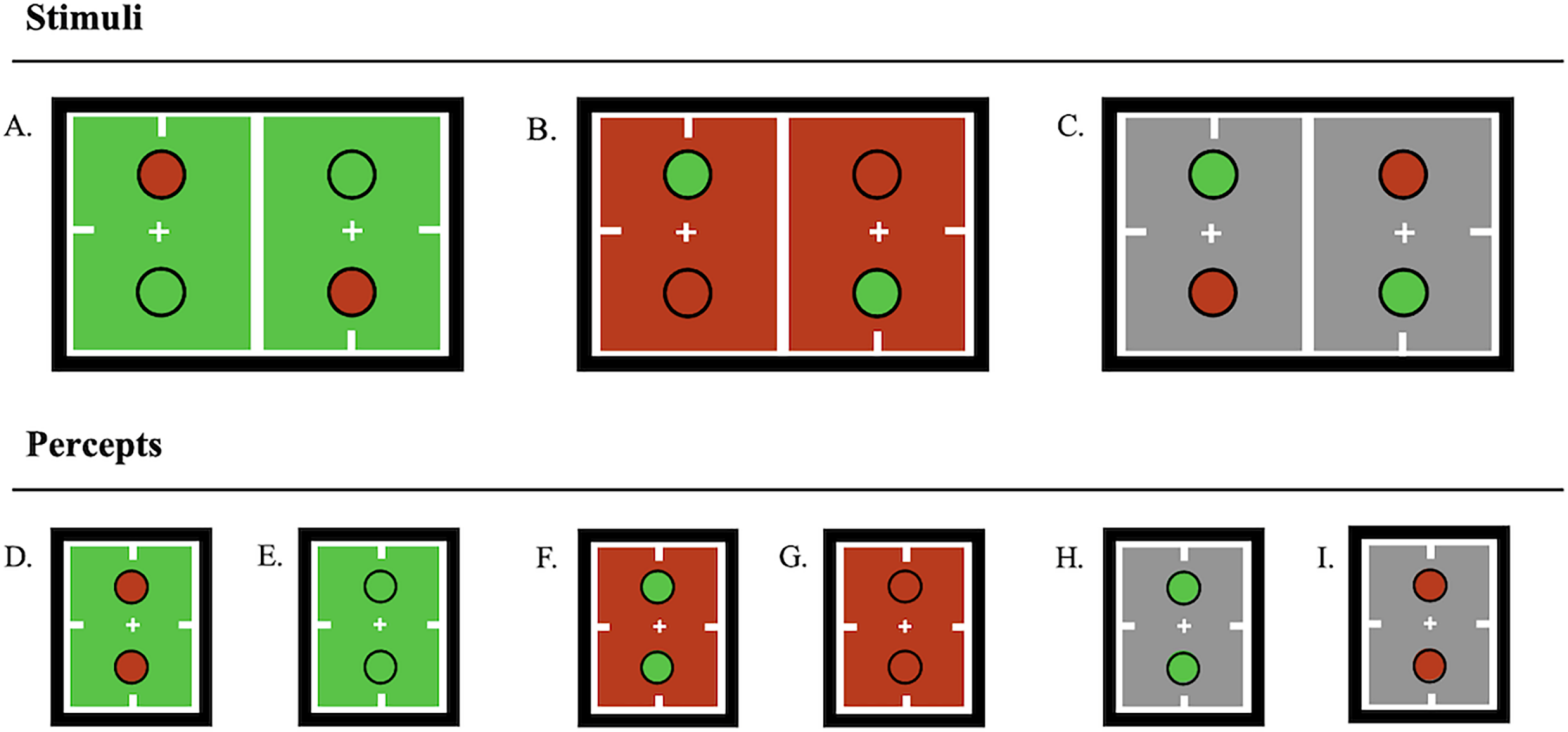
Stimuli and measured percepts for Experiment 1. (**A**) Rivalrous disks within a stable green-appearing background. (**B**) Rivalrous disks within a stable red-appearing background. (**C**) Rivalrous disks within a stable gray-appearing background. (**D**, **E**) Measured percepts for the stimulus depicted in A. (**F**, **G**) Measured percepts for the stimulus depicted in B. (**H**, **I**) Measured percepts for the stimulus depicted in C. (**D**/**F**) Difference-enhanced percepts. (**E**/**G**) Similarity-enhanced percepts.

All stimulus arrays shared the same arrangement, while chromaticity and rivalry status were varied. Each array featured two rivalrous regions with 1.5° diameters. Rivalrous regions were spatially stacked, such that the top disk was located 1.5° above fixation, and the bottom disk was located 1.5° below fixation (Figures 3A–C). Because rivalry dynamics can exhibit persistent, location‑specific biases within the visual field—particularly for the eye of presentation (sensory eye dominance)—the displays were vertically symmetric, and analyses emphasize within‑observer ordinal predictions, which mitigate location bias in between‑condition contrasts (Dieter, Sy, & Blake, 2017). All stimuli were presented inside 4.5°-by-4.5° fusion boxes with Nonius lines. Fusion box edges were at a chromaticity metameric to the equal-energy spectrum and were in luminance contrast (Y = ∼25 cd/m^2^) relative to their interior background. All stimuli had two vertically oriented rivalrous regions with dark annuli (Y = ∼0.1 cd/m^2^), increasing the total visual angle of disks with annuli to 1.75°. Annuli were included to aid fusion, control for chromatic edge contrast, and separate background and disk rivalrous regions in Experiment 2. Nonrivalrous regions, such as the fixation point, Nonius lines, fusion boxes, and annuli, remained constant across all trials.

The chromaticities of color regions for all conditions were set at [L/(L + M), S/(L + M)] values of [0.62, 0.30], referred to as “green,” or [0.71, 0.30], referred to as “red” (MacLeod & Boynton, 1979). The achromatic value, referred to as “gray,” was [0.665, 1.0]. Note that the unit of [S/(L + M)] is arbitrary and was set to 1.0 for equal-energy-spectrum “white.” Red-, green-, and gray-appearing chromaticities were presented at moderate photopic levels (∼15 cd/m²) to avoid the nonlinear luminance–perception distortions and photopigment bleaching that can occur at higher luminance levels (Kaiser & Boynton, 1996; Stockman & Sharpe, 2006).

### Experimental protocol

The experimental protocol was identical for both experiments. The trial order was randomized for each observer on each experimental day. Prior to each session and after any breaks, observers underwent 5 minutes of dark adaptation to stabilize cone sensitivity and minimize variability in retinal responsiveness (Shapley & Enroth-Cugell, 1984). Instructions were displayed on the screen using images to indicate target percepts. Text instructions indicated the gamepad buttons corresponding to each target percept. During each trial, observers were instructed to press and hold buttons on a gamepad for the duration they experienced each measured percept (described below) and to withhold button presses for all percepts not indicated by the instructions, including partially resolved or piecemeal percepts.

Total dominance durations were calculated by taking the average dominance duration of each measured percept for each of 3 experimental days. Standard errors of the mean were calculated using the mean total dominance durations for each of the 3 days to estimate between-day variance in order to assess the reliability of the results. All subjects participated in both experiments, requiring them to come into the lab for 6 days. The first 3 days entailed a vision screening and three HFP sessions, one on each day. On the third day, subjects also practiced both experiments (these data were not analyzed). Data were analyzed from the final 3 days of the experiment, in which observers completed the same trials in a different random order on each of the 3 days. Each trial began with an instruction screen that provided a visual cue for which button to press for each measured percept. Measurements began following the initial 10 seconds and continued for 60 seconds to reduce the possible impact of differential adaptation between the two eyes from the onset of the ISR phase and potential onset effects (Carter & Cavanagh, 2007).

## Experiment 1

Peiso and Shevell (2020) observed experimental conditions that diminished similarity enhancement (grouping) but increased difference enhancement. This raises the following question: Can pooled divisive normalization account for these results? Experiment 1 was designed to test whether nonrivalrous, chromatically stable background signals would pool with similar signal components from rivalrous regions and, in doing so, influence perceptual resolution. According to the normalization model, the visual signal is reweighted such that less pervasive signals are more likely to dominate during rivalry. In practical terms, when a red/green rivalrous disk is presented on a stable green background, the model predicts that divisive normalization will enhance the difference between the signals—making the red percept more likely. To rule out the possibility that any observed effect is simply due to chromatic contrast rather than a chromatically tuned normalization process, conditions with a gray background were also included. Because these predictions follow directly from applying the model to the stimuli, the full computational derivation is provided immediately after the Stimuli and Procedure section.

### Stimuli and procedure

Experiment 1 featured stimuli with nonrivalrous, dichoptically stable backgrounds (Figures 3A–C). Three experimental conditions were included, each with two types of measured percepts: (a) both disks resolved as green and (b) both disks resolved as red. For conditions with chromatic backgrounds (Figures 3A, 3B), disks resolved as the same color as their background were considered similarity-enhanced (Figures 3E, 3G), and disks resolved as a contrasting color to their background were considered difference-enhanced (Figures 3D, 3F). Stimulus C had a neutral gray-appearing background (Figure 3C) with the same red/green rivalrous disks. Measured percepts (Figures 3H, 3I) for stimulus C were considered a baseline for resolving rivalrous disks as red or green.

### Model predictions for Experiment 1

Within the pooled‐normalization model of interocular grouping presented here, increasing the spatial prevalence of a particular chromatic signal (e.g., a green background) produces a characteristic staircase‐like modulation in perceptual outcomes. For a stimulus with a green background, neurons tuned to the background color (green) are pooled with those responding to the green component of the rivalrous stimulus, and the pool of green-responsive neurons mutually imposes divisive normalization. This shared normalization imposes a stronger attenuation on the green drive, thereby lowering the probability that observers will perceive the rivalrous region as green. This prediction follows directly from the normalization step (Equations 1 and 2). Consider a trial with a stable green background (Figures 3A, 3D, 3E). First, the normalized drive for red is calculated by summing over the red pixels in rivalrous regions only, $f\left(\bar{x}\right)\circ S_{R}\left(\bar{x}\right)$, and dividing it by the sum over all red pixels in the stimulus, $\Sigma S_{R}\left(\bar{x}\right)$, and then adding random channel noise that is proportional to the signal (η*_R_*). For this stimulus, the normalized drive for red will be a multiplier of value 1, before adding random noise fluctuations. Next, the normalized drive for green is calculated the same way; the difference is in the denominator, which includes all background pixels, in addition to the green pixels within the rivalrous regions. This would set the normalized gain multiplier for green to 0.069 before adding random noise. Finally, these two normalized signals are compared via the recursive winner-take-all maxing function (Equation 5). As a result, the red signal dominates most of the time, though adaptation (Equations 6–7), noise (Equation 3), and stochastic gain fluctuations (Equation 4) occasionally can allow green to be perceived. With a neutral (gray) background, the model predicts no bias toward either color, yielding equal probabilities for red and green percepts (see Supplementary Table S1).

### Experiment 1 results

**Figure 4.**
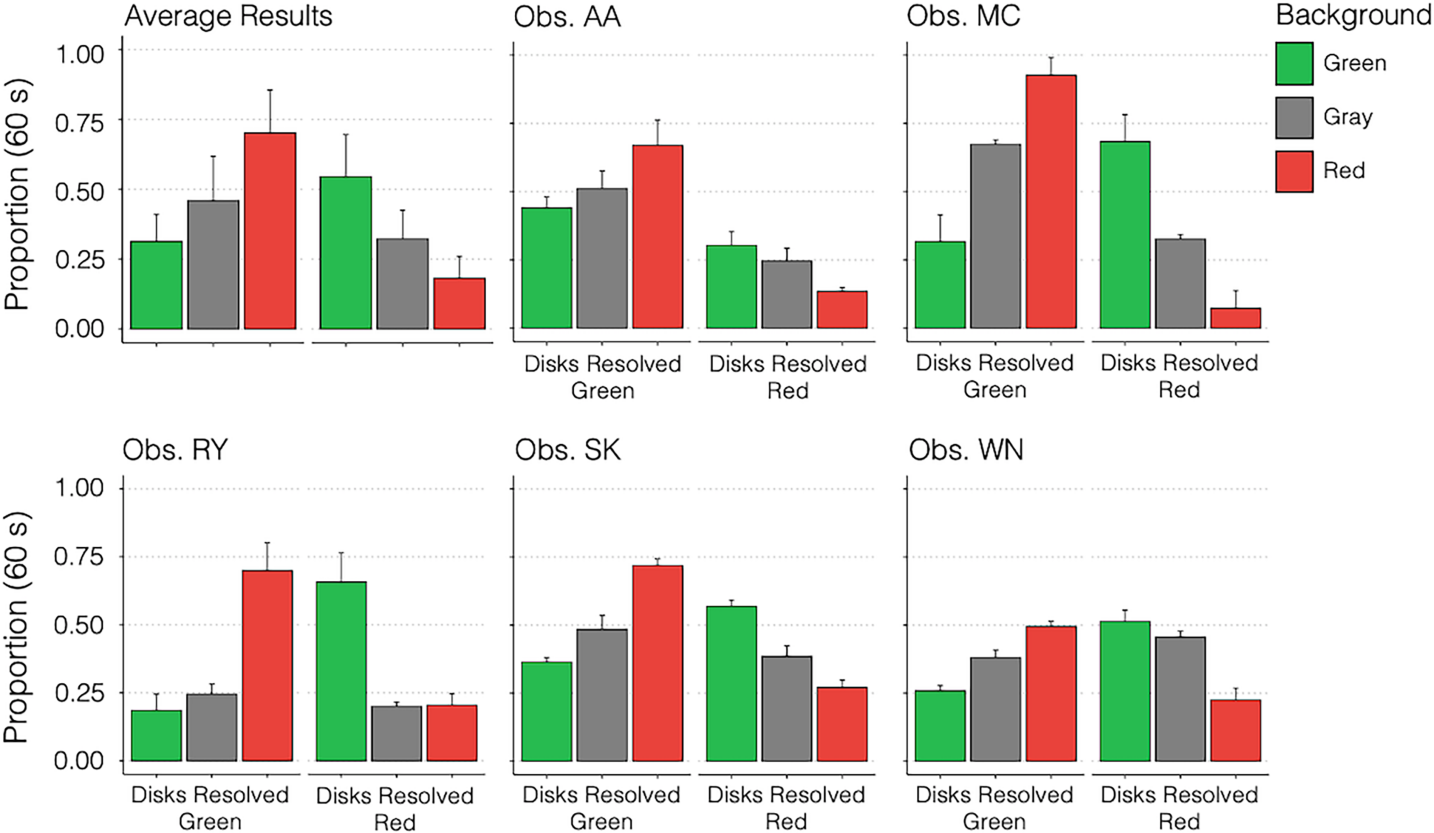
Average results and individual measurements for each of five observers. The vertical axis is the proportion of a 60-second trial in which each percept was seen. Bottom horizontal axis groups the results by perceived disk color (left bars: green disks; right bars: red disks). Bar color indicates the background color for each measurement. Error bars indicate standard error of the mean for measurements taken across 3 days. Top left graph represents the group mean results, and error bars indicate the standard deviation across subjects.

Experiment 1 was designed to test a precise prediction of relative dominance durations for red/green rivalrous disks. Specifically, disks should resolve most frequently as color-contrasted against their background and least frequently as the same color as the background. Gray backgrounds were expected to elicit intermediate dominance durations. When grouped by resolved disk color, observer data should resemble two “staircases,” one increasing and the other decreasing left to right. Figure 4 presents both individual‐observer staircases and the group mean.

At the group level (Figure 4, upper left), the staircase pattern is evident. Green-disk resolutions rose from 0.313 (*SD* = 0.098) on a green background to 0.459 (*SD* = 0.159) on a neutral background and to 0.702 (*SD* = 0.154) on a red background. By contrast, red-disk resolutions fell from 0.545 (*SD* = 0.151) on a green background to 0.323 (*SD* = 0.103) on a neutral background and to 0.182 (*SD* = 0.078) on a red background. The results averaged across observers also produce the predicted “staircases.”

For each observer, their two staircases (green‐disk and red‐disk resolutions) were considered a “success” if they followed the model's relational prediction (green background: green < neutral < red; red background: red < neutral < green). Under the null hypothesis, all six possible orderings are equally likely; the chance of any one staircase matching the prediction is 1/6. Across five observers (10 staircases), 9 staircases conformed to the prediction. A one‐tailed (directional, a priori) binomial test on *k* = 9 successes out of *n* = 10 yields *p* < 0.001. A variant binomial test restricts “success” to both staircases matching the prediction for a given observer (chance = (1/6)²); with 4/5 such successes, the one‑tailed binomial is *p * < 0.001, leading to the same conclusion. This nonparametric approach directly evaluates the predicted ordinal structure; multiple pairwise tests (e.g., green vs. neutral, neutral vs. red) do not capture that relation as succinctly or robustly.

These results offer clear support for the impact of chromatic background context on perceptual dominance durations, in line with predictions from a chromatically tuned divisive normalization model. The next experiment considers the potential role of chromatic edge contrast on the observed results.

### Experiment 1 model results

**Figure 5.**
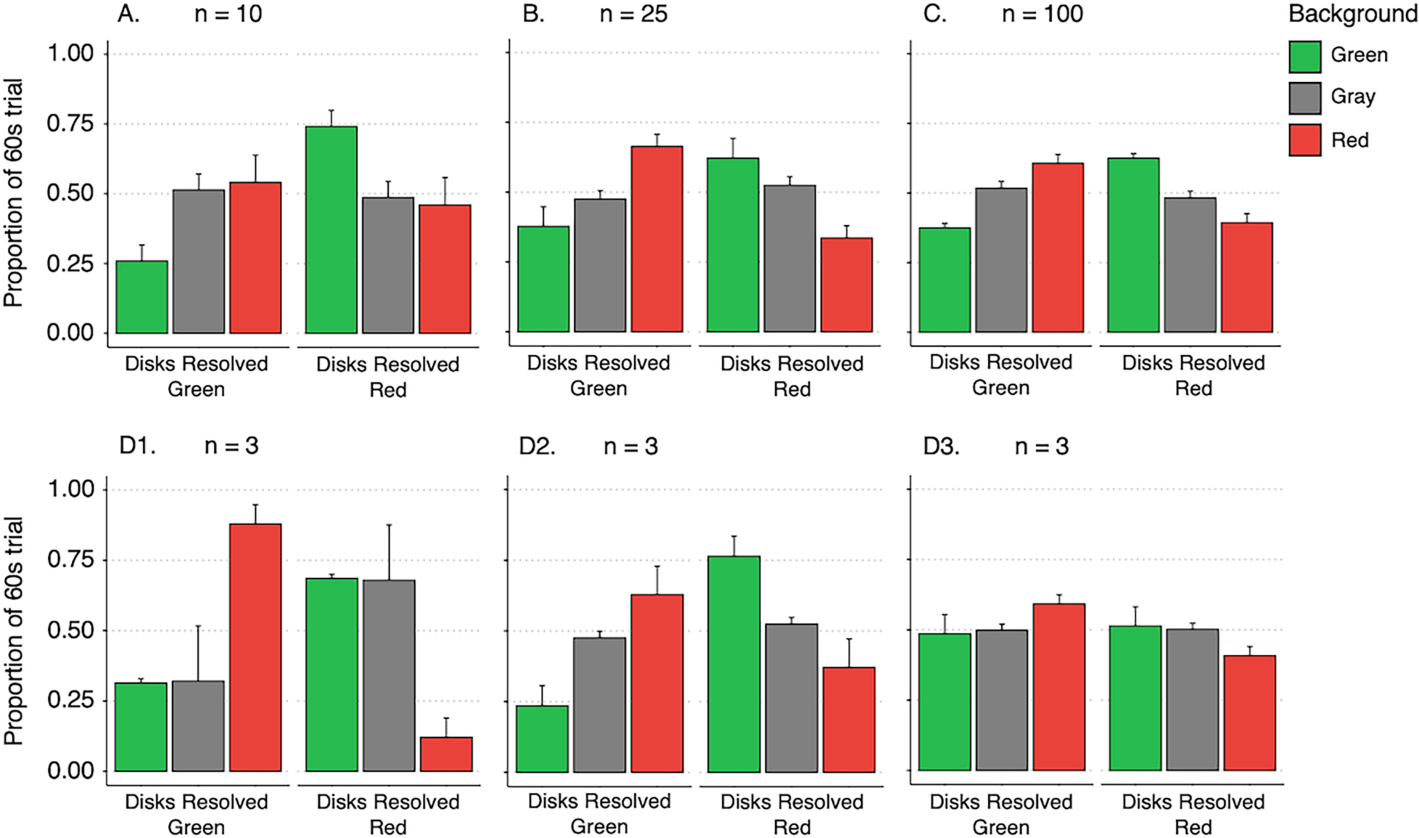
Simulated results for Experiment 1. (**A**–**D**) Mean proportion of total dominance time for green (left clusters) and red (right clusters) percepts under gray, green, and red backgrounds computed for different trial numbers. Top row: The mean proportion of total dominance time for *n* = 10 (**A**) *n* = 25 (**B**), and *n* = 100 (**C**) independent simulated trials. Bottom row: The mean proportion of total dominance time for three instances of *n* = 3 (**D**) independent simulation runs. Bars are color-coded by background condition and grouped by perceptual outcome—green-disk resolutions on the left and red-disk resolutions on the right. Error bars denote the standard deviation from the mean across *n* runs.

All model parameters were set based on biologically motivated values, leaving no free parameters for fitting. Alternative combinations of these parameters are explored. Here, extreme-valued parameters reveal the trade-offs and breaking points of the model (see Supplementary Materials). Simulated results for Experiment 1 are consistent with the model's predictions. These results produce the characteristic staircase-like pattern empirically observed in Figure 4. Figure 5 illustrates how the characteristic staircase pattern of dominance durations emerges and stabilizes as the number of simulated trials increases. The top row in Figures 5A–C plots the mean proportion of total dominance time for green-disk and red-disk percepts under green, neutral (gray), and red backgrounds for *n* = 10, 25, and 100 independent runs. By *n* = 25, the mean proportions lie within approximately 2% of the *n* = 100 values, and by *n* = 100, the staircase-like bias has effectively stabilized. The bottom row (Figure 5D) shows three instances of an *n =* 3 experiment to highlight the model's intrinsic stochasticity: With so few trials, estimates become noisy and occasionally nonmonotonic. Notably, this degree of variability mirrors that seen in the human observers (Figure 4), where results were likewise averaged over 3 measurement days (*n* = 3).

Perceptual alternations in rivalry depend on stochasticity in addition to adaptation: Moderate internal fluctuations are needed to drive state transitions without yielding perpetual lock‑up or erratic, unstructured switching (demonstrated in neural‑competition and attractor models) (Kim et al., 2006; Moreno Bote, Rinzel, & Rubin, 2007; Shpiro et al., 2009; Wilson, 2003), and these effects are especially evident under weak or ambiguous stimulation (Brascamp et al., 2006).

Physiologically, single neurons often exhibit near‑Poisson trial‑to‑trial variability that is “quenched” (reduced) at stimulus onset, and population reliability improves further when pooling across weakly correlated neurons; moreover, attention reduces shared variability in area V4, all of which supports modeling a modest, often sub‑Poisson population‑level noise term rather than matching single‑unit variance (Averbeck, Latham, & Pouget, 2006; Cohen & Maunsell, 2009; Churchland et al., 2010; Mitchell, Sundberg, & Reynolds, 2009). Because perceptual reports reflect the readout of distributed activity across multiple visual areas rather than a single neural population, we parameterize noise at the level of an integrated population/decision variable, not single-unit spiking (Gold & Shadlen, 2007; Liu et al., 2020).

In this framework, the noise term captures perceptual-level instability that interacts with adaptation, multiplicative gain, and contextual modulation to resolve ambiguous input. We implement noise as a truncated normal (mean = 0, σ = 1) bounded at ± 0.35; after truncation, the effective standard deviation is ≈0.2 (∼20%), providing enough stochasticity to destabilize ongoing percepts while preserving the signal structure needed for contextual biases and coherence. A sensitivity analysis shows the expected qualitative regimes: High-noise settings produce unstable, flicker-like alternations, whereas low-noise settings suppress switching and approach determinism; simulations demonstrating these effects are provided in the Supplementary Figures S2 and S3.

A multiplicative gain factor, *g*, was modeled as a constant (*g* = 1.3) to simulate a bias that amplifies the selected signal by 30%—a value consistent with estimates of gain modulation observed in V4 neurons (McAdams & Maunsell, 1999; Reynolds & Heeger, 2009). At each 4 Hz update—consistent with rhythmic sampling of multiple/overlapping stimuli—the gain is stochastically assigned to one channel, with *p* = 0.55 for the currently stronger signal and *p* = 0.45 for the weaker, implementing a weak biased-competition dynamic; this parametrization operationalizes the idea that attention biases competition toward the dominant feature and stabilizes perception (Davidson et al., 2018; Dieter & Tadin, 2011; Li, Rankin, Rinzel, Carrasco, & Heeger, 2017; Ling & Blake, 2012; Re et al., 2019). Sensitivity analyses (Supplement) show that varying either *g* or the gain bias probability modulates the magnitude of the staircase effect but preserves its ordinal pattern (see Supplementary Figures S4–S7).

Realistic alternation dynamics in binocular rivalry require that dominant signals slowly attenuate over time while suppressed signals gradually recover. To capture these processes, two biologically grounded time constants are used. The adaptation time constant, τ*_D_* = 2.5 seconds, was chosen to match estimates from higher visual areas like V4, where gain control and feature tuning exhibit prolonged changes over time (Kohn, 2007; Mei, Dong, & Bao, 2017). Chromatic adaptation operates across multiple spatial and temporal scales relevant to perceptual stability (Werner, 2014), consistent with seconds-scale τ*_D_* used here. Recovery of suppressed signals is modeled with a shorter time constant, τ*_R_* = 1.5 seconds, consistent with prior computational models of perceptual bistability that implement asymmetric dynamics to allow the reemergence of suppressed percepts (Shpiro et al., 2009; Wilson, 2003). These values reflect empirically observed latencies in neural response modulation and are critical for producing alternation rates and dominance durations that align with human rivalry data. Although adaptation and recovery are conceptually continuous, those dynamics update at 100 Hz to capture their time courses efficiently and a base integration time step of 1 ms (1 kHz) to balance biological realism with computational tractability. Sensitivity analyses show that short rate constants that drive nearly instantaneous decay result in a slightly attenuated background-dependent staircase (see Supplementary Figure S8), but alternation dynamics are enhanced. Similarly, long decay constants also produce the characteristic staircase, but the alternation dynamics are substantially suppressed (see Supplementary Figure S9). The influence of extreme values for the recovery constant (τ*_R_*) showed a similar pattern of intact average results but disrupted trial dynamics (see Supplementary Figures S10–S11).

Together, these results establish that pooled divisive normalization—augmented by physiologically plausible noise, gain dynamics, and adaptation/recovery time constants—is sufficient to generate the precise staircase-like pattern of perceptual dominance observed in Experiment 1. These parameters can be adjusted to fit individual differences, which are commonly observed in rivalry experiments (e.g., Brascamp, Becker, & Hambrick, 2018; Brascamp, Qian, Hambrick, & Becker, 2019; Gallagher & Arnold, 2014; Pettigrew & Miller, 1998).

## Experiment 2

**Figure 6.**
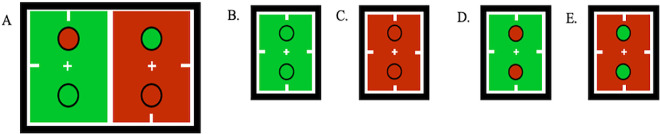
Stimuli and measured percepts for Experiment 2. (**A**) Stimulus: Rivalrous disks within a rivalrous background context. (**B**, **C**) Measured similarity-enhanced percepts for stimulus A. (**D**, **E**) Measured difference-enhanced percepts for stimulus A.

To rule out the possibility that the dominance of difference-enhanced percepts in Experiment 1 is explained by a saliency mechanism acting on the chromatically contrasted monocular images, Experiment 2 substituted the neutral gray background of Experiment 1 with dichoptically rivalrous red and green backgrounds (Figure 6A). Black annuli were retained such that disk edges remained in luminance contrast. Similarly, to hold chromatic contrast identical to the stimuli in Experiment 1, each eye's image contained one chromatically contrasted disk. Under these conditions, each disk's drive is divided by the same pooled signal, so red and green inputs are equally normalized. If chromatic edge contrast alone were responsible for the difference enhancement in Experiment 1, observers would again report disks appearing in contrast to their background in Experiment 2. Conversely, the divisive normalization model here predicts a robust similarity enhancement—disks matching the background hue—because the normalized drives are identical, apart from stochastic noise and gain fluctuations. Calculations of these normalized signals are provided in Supplementary Table S1.

### Experiment 2 stimuli

The stimuli in Experiment 2 were similar to those in Experiment 1, except that the dichoptically stable nonrivalrous backgrounds in Experiment 1 were replaced with chromatically rivalrous backgrounds in Experiment 2 (Figure 6A). Each trial had four measured percepts since the background and disk regions could each resolve as red or green (e.g., Figures 6B–E). Percepts were categorized as similarity-enhanced if the disks resolved to be the same color as the background (Figures 6B, 6C) or difference-enhanced (Figures 6D, 6E) if the disks resolved to be a different color than the background. Observers completed three sessions on separate days; dominance durations for each percept type were measured in 60-second trials and averaged across days.

### Experiment 2 results

**Figure 7.**
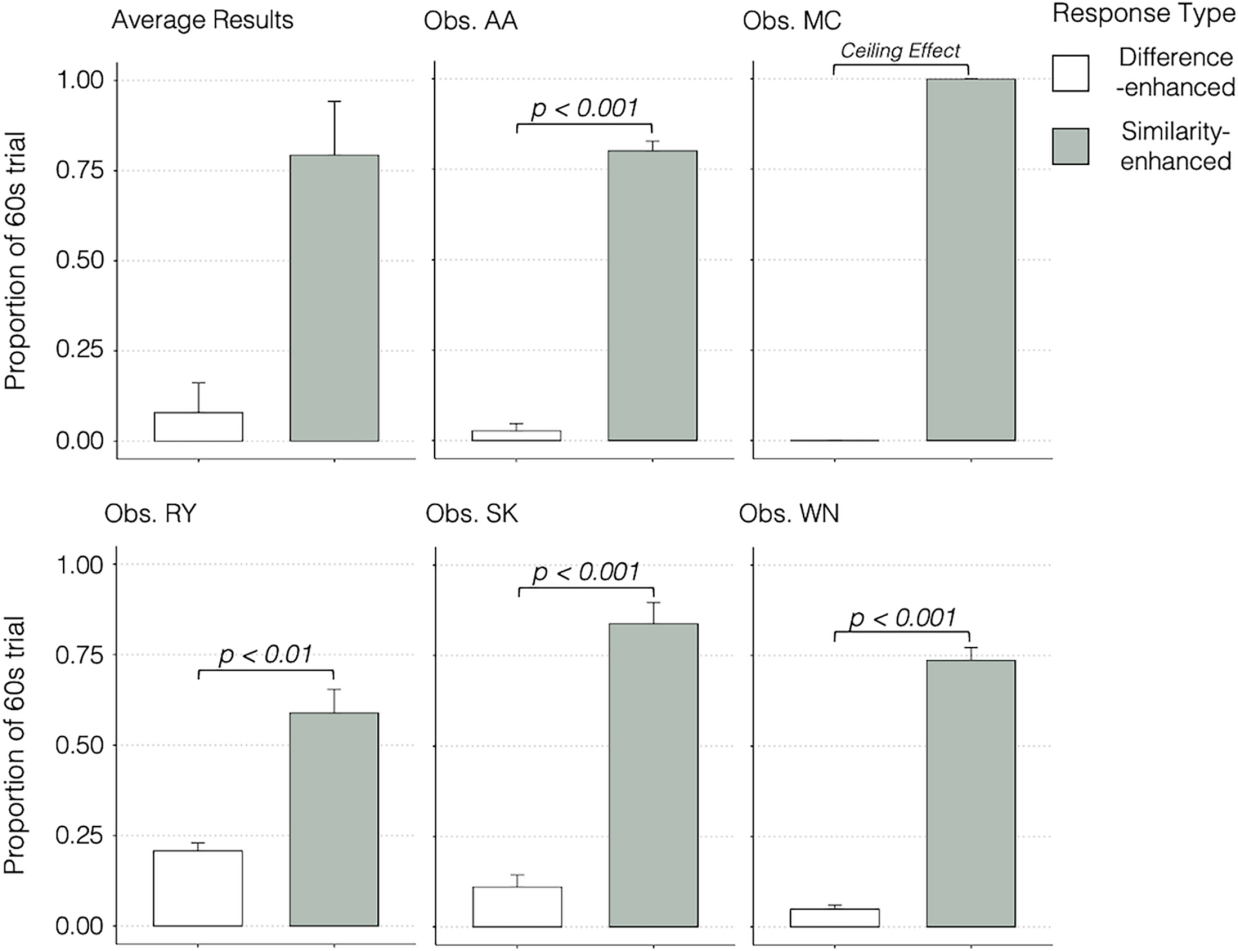
Average results and planned contrasts for five observers. The vertical axis is the proportion of a 60-second trial in which each percept was seen. The horizontal axis indicates the response type (“Difference-Enhanced” or “Similarity-Enhanced”). Top left plot shows average results, and error bars indicate the standard deviation across subjects. Brackets indicate a significant contrast.

As predicted by the normalization model, similarity-enhanced percepts overwhelmingly dominated perception. At the group level (Figure 7, upper left), similarity-enhanced percepts occupied 0.793 (*SD* = 0.149) of total dominance time, whereas difference-enhanced percepts accounted for only 0.079 (*SD* = 0.083). Error bars in Figure 7 denote the standard error of the mean across days. A planned contrast—one per observer—confirmed this effect: Four observers showed a significant similarity enhancement bias (*p* < 0.01), and the fifth (MC) displayed a ceiling effect with no reported difference-enhanced percepts at all. These results closely follow the prediction that equal-sized normalization pools should produce similarity-enhanced outcomes, demonstrating that chromatic edge contrast alone cannot account for the difference enhancement seen in Experiment 1. Because both chromatic inputs draw on the same normalization pool, the model presented here never produces difference‐enhanced percepts under these conditions by design, so that outcome was not simulated. The small residual difference enhancement seen in a few observers may instead reflect local iso‐feature suppression processes in early visual cortex (e.g., Li, 1999) that transiently amplify contrast signals before they enter the pooled normalization stage.

## Discussion

This study explored a chromatically tuned divisive normalization model to account for experimentally measured biases in the perceptual resolution of neural ambiguity during ISR. The findings provide clear evidence that the chromatic context of the background influences the dominance durations of percepts, in line with predictions derived from a model implementing chromatically tuned divisive normalization. Specifically, the results of Experiment 1 demonstrate that difference-enhanced percepts are more likely to dominate perception when chromatically contrasted rivalrous regions are presented against stable backgrounds. It was hypothesized that difference-enhanced percepts would dominate perception when rivalrous stimuli were presented against a background chromatically congruent with one of the rivalrous chromaticities. This prediction emerges from the proposed framework; specifically, the neural response to the chromatic background is pooled with and attenuates the congruent component of the rivalrous signal. This process, in turn, allows the difference-enhanced representation to dominate perception.

Experiment 2 was designed as a critical test between the present framework and a simple saliency account. Each monocular image contained a chromatically contrasted disk, so at the monocular level, chromatic contrast was identical to Experiment 1. At the binocular level, both the central disks and the chromatic backgrounds were in rivalry. Under these conditions, the two competing neural pools should have equal divisive strength, and this symmetry biases perception toward similarity enhancement. The data align with this prediction: Similarity-enhanced percepts dominated perception in Experiment 2. Since the saliency of monocular images was constant across experiments, similarity-enhanced percepts are difficult to reconcile with a saliency-based explanation driven by local feature contrast.

### Individual differences

Robust individual variation in binocular‑rivalry dynamics is well documented and appears to have structured components: Large‑sample work indicates trait‑like stability and points to two partly independent factors—a feature‑specific factor linked to the treatment of interocular conflict and a more general factor shared with other bistable phenomena (Brascamp et al., 2018; Brascamp et al., 2019). These differences manifest in both temporal metrics (e.g., dominance durations, switch rate) and qualitative states (e.g., mixed/piecemeal percepts) (Gallagher & Arnold, 2014; Pettigrew & Miller, 1998). Potential contributors include sensory eye dominance and retinotopic/location biases (Dieter et al., 2017), age (Arani, van Ee, & van Wezel, 2019), attentional/working memory influences (Jensen, Gips, Bergmann, & Bonnefond, 2014; Scocchia, Valsecchi, Gegenfurtner, & Triesch, 2014), conflict‑sensitive gating in visual cortex (Katyal et al., 2018), and genetic factors (Shannon, Patrick, Jiang, Bernat, & He, 2011).

Individual differences are present here as well, but the experiments are powered for within‑observer ordinal tests; each observer serves as the control for their own comparisons, so between‑observer variability is not interpreted quantitatively.

The predicted staircase pattern of results was found for each observer despite the steepness of the rises and falls that vary across individuals (e.g., MC's nearly ceiling‐level green‐disk dominance on a red background vs. AA, SK, and WN's more graded transitions). As expected, Figure 5D demonstrates that, with only three trials (*n* = 3), stochastic noise alone can generate run‐to‐run variability of similar magnitude. In Experiment 2, four of five observers exhibited a statistically significant similarity enhancement bias (*p* < 0.01; Figure 7). The fifth observer, MC, produced a ceiling effect—never reporting difference‐enhanced percepts—again mirroring the model's deterministic absence of difference-enhanced percepts when normalization pools are balanced. Across both experiments, these individual difference profiles fall within the spread of the model's stochastic simulations (Figure 5), echoing the idiosyncratic alternation dynamics that can emerge from the same core divisive‐normalization framework.

### Comparison with similar models

Many existing models of binocular rivalry primarily focus on rivalry dynamics, such as stochastic switching (e.g., Brascamp et al., 2006; Freeman, 2005; Kim et al., 2006; Moreno-Bote, Rinzel, & Rubin, 2007), the combined effects of noise and adaptation (e.g., Lankheet, 2006; Lehky, 1988; van Ee, 2009; Wilson, Blake, & Lee, 2001; Wilson, 2003), or attentional influences on visual awareness (e.g., Brascamp & Blake, 2012; Dieter et al., 2016; Hancock & Andrews, 2007; Lee & Blake, 2004; Li et al., 2017; Ling & Blake, 2012; May & Zhaoping, 2022; Said & Heeger, 2013). While these models may explain how perceptual switches can occur over time, they do not address how specific chromatic percepts dominate during rivalry. The model here integrates adaptation, noise, and multiplicative gain but shifts focus to perceptual selection—the determination of which percept becomes dominant in a given context. Divisive normalization enables this model to estimate signal strength in stimuli of equal luminance, refining how percept dominance is predicted. Unlike traditional models, which rely on mutual inhibition and winner-take-all competition (Lee & Blake, 1999), the model here incorporates competition based on the relative signal strength of competing neural representations influenced by feature-tuned divisive normalization. In this framework, the competition is not solely determined by inhibitory dynamics but also by contextual and intrinsic factors. Like Ling and Blake (2012), the model uses divisive normalization as a key mechanism regulating neural competition during rivalry. Whereas Ling and Blake emphasize spatially defined normalization pools, the model here uses feature-tuned pools and may incorporate object-based, serially dependent biases. This extends the normalization framework to capture context-dependent, temporally sustained perceptual stability in chromatic rivalry.

The V1 saliency hypothesis yields the same prediction for Experiment 1 as the proposed model. Here, a V1 saliency map could explain the perceptual dominance of difference-enhanced percepts observed in Experiment 1. Saliency, defined here as the tendency of a location to attract bottom-up selection in the absence of strong top-down guidance, is best probed by minimizing a priori knowledge and cues about target position or features (Zhaoping, 2014). In the V1 saliency framework, contextual iso-feature suppression among V1 neurons is proposed to underlie visual search by enhancing the salience of feature singletons at a given location (Li, 2002). This framework has been extended to dichoptic search displays, where intraocular iso-feature suppression in V1 enhances the salience of an eye-of-origin singleton and guides local selection of the attended or gazed-at location before binocular combination (Zhaoping, 2008; Zhaoping, 2012). Iso-feature suppression in V1 arises from monocular neurons mutually suppressing nearby neurons with similar feature tuning (Li, 1999; Li, 2002; Zhaoping, 2014), and this lateral interaction pattern can give rise to an effective computation akin to divisive normalization. Although the V1 saliency hypothesis and the present model share a local motif of mutual suppression between similarly tuned neurons, the frameworks differ in both objective and predictions: The V1 saliency hypothesis is formulated to explain how local feature contrast drives attentional and oculomotor selection, whereas the current model addresses how the neural ambiguity induced by chromatically rivalrous dichoptic stimuli at a known and unchanging location is perceptually resolved. Stated simply, the V1 saliency framework seeks to explain *where* in space attentional resources should be allocated, and the present work seeks to explain *what* an observer will perceive if they are presented with two incompatible, isoluminant chromatic stimuli. Furthermore, these frameworks also differ in how mutual suppression among similarly tuned neurons biases perception. The present model pools signals supporting each “color hypothesis” across both eyes and both rivalrous regions, so that perception depends on a comparative operation between competing normalization pools rather than on the local suppression itself.

In Experiment 2, however, a V1 saliency framework does not yield the same prediction as the present framework. A V1 saliency map that computes local feature contrast would assign high salience to the rivalrous region in both monocular inputs but would not, on its own, reduce the neural ambiguity evoked by chromatic rivalry. In this sense, a saliency mechanism does not offer a clear explanation for the perceptual dominance of a uniform, low-salience percept. By contrast, in the present model, this behavior arises from how feature-tuned normalization pools are constructed: Signals supporting the same color (e.g., red or green) contribute to a shared pool spanning both rivalrous regions and both eyes, and the symmetry of these pools in Experiment 2 biases perception toward similarity-enhanced, uniform percepts. Furthermore, the results reported here suggest activity from higher visual areas, such as V4, where the representation of a color percept is distinguishable from stimulus chromaticity (Kim et al., 2020; Liu et al., 2020). Neural competition and attentional modulation are also more pronounced in areas such as V4, with larger receptive fields than the primary visual cortex (Kastner & Ungerleider, 2001; Luck, Chelazzi, Hillyard, & Desimone, 1997). Evidence that attention helps resolve rivalry (Dieter & Tadin, 2011), together with demonstrations that chromatic percepts are represented in extrastriate cortex (e.g., V4; Liu et al., 2020), points to feature-tuned pooling beyond V1. Early mechanisms such as exogenous saliency (Zhaoping, 2016) and iso-feature suppression in V1 (Li, 1999; Li, 2002) are likely local contributors, but the observed pattern implicates extrastriate, feature-tuned pooling.

Other types of percepts have been reported for related dichoptic stimuli. In one such experiment using dichoptic color images, disparate monocular images of compatible three-dimensional object surfaces evoked stable, dichoptically completed transparent percepts rather than rivalry (Zhaoping & Meng, 2011). These results were interpreted as reflecting an object- and depth-based interpretation of the dichoptic inputs. By design, the chromatic patches used here were strictly isoluminant and lacked such object and depth cues, so the present work focuses on perceptual selection during chromatic rivalry. The model does not address transparency or surface completion.

### Future directions

The tuned divisive normalization framework here successfully accounts for both similarity- and difference-enhanced percepts evoked during chromatic ISR and suggests several avenues for further exploration. First, systematically varying stimulus size—beyond the relatively small disks used here—may reveal how normalization pools scale with larger or more complex visual fields, potentially affecting the balance between grouping and segmentation processes. Second, examining how individual observer traits (e.g., attentional strategies, neural tuning) influence parameter settings could clarify some of the variability seen in rivalry experiments. Attention was not a considered factor for the experiments presented herein; however, the task did require focal spatial attention. While attentional focus inherently fluctuates over time, the observers’ goal of focal attention was held constant across experiments. Future work that directly manipulates or indexes attentional focus, via increased cognitive load or with an eye-tracker, could reveal how the visual system flexibly adjusts its divisive normalization computations and navigates the trade‐off between similarity and difference enhancement under different task demands. It may be the case that under high‐load conditions, when efficiency is paramount, similarity‐enhanced grouping dominates perception, regardless of chromatic context.

## Conclusions

Classic rivalry models typically rely on mutual inhibition and adaptation to explain alternations (e.g., Freeman, 2005; Wilson, 2003), but these frameworks cannot fully account for interocular grouping or difference enhancement. More recent accounts, such as Said and Heeger (2013), incorporate divisive normalization to explain cross-orientation suppression and binocular competition, yet still do not directly address grouping or difference enhancement. In comparison, the present findings indicate that a single, flexible mechanism—tuned divisive normalization—can reconcile these seemingly antagonistic processes. Rather than serving solely as a mutual inhibition mechanism, divisive normalization in the model here rebalances signals within competing populations, enabling both grouping and segmentation. Far from contradicting similarity‐based grouping, the model demonstrates that linking similarly tuned neurons (Barlow, 1981) through normalization pools can flexibly yield either difference‐ or similarity‐enhanced perceptual resolution.

## Supplementary Material

Supplement 1
