# Supplementary material for: Perceptual resolution of ambiguity: A divisive normalization account for both interocular color grouping and difference enhancement
Source: J Vis. 2026 Jan 13;26(1):8. doi: 10.1167/jov.26.1.8 (PMC12811879; doi:10.1167/jov.26.1.8)
Supplement: Supplement 1 [file jovi-26-1-8_s001.pdf]

Supplemental Material

Model Inputs

The model is driven by two stimulus-defined signals, $S_R(\bar{x})$ and $S_G(\bar{x})$, which are obtained by summing the red- and green-valued pixels individually across the stimulus images of both the left and right eyes. These signals are convolved with a spatial filter, $f(\bar{x})$, to isolate rivalrous regions from dichoptically-stable regions. This quantity is then normalized by $S_R(\bar{x})$ and $S_G(\bar{x})$ over all $\bar{x}$ (Eqs. 1.1 and 1.2) to produce normalized outputs, $\hat{S}_R$ and $\hat{S}_G$. This calculation is done for the stimuli used in experiments 1 and 2 (see Table S1). An additive stochastic term η (Eq. 1.3) is independently applied to each normalized signal. The stochastic parts of the model are addressed in the next section, which explores the model's sensitivity to tunable parameters, such as η.

Normalization Step

A crucial point of this exercise is to show that $S_R(\bar{x})$ and $S_G(\bar{x})$ are not tunable model parameters; instead, they are entirely specified by the stimulus chromaticities and the spatial filter, $f(\bar{x})$. Likewise, $\hat{S}_R$ and $\hat{S}_G$ are largely deterministic for each stimulus. For each experiment's stimuli, the deterministic part of the normalized red and green signals is computed by

$$\hat{S}_R = \frac{\sum_{\bar{x}} f(\bar{x}) \circ S_R(\bar{x})}{\sum_{\bar{x}} S_R(\bar{x})} \quad \text{and} \quad \hat{S}_G = \frac{\sum_{\bar{x}} f(\bar{x}) \circ S_G(\bar{x})}{\sum_{\bar{x}} S_G(\bar{x})}.$$

Spatial Filtering of Raw Signals ($f(\bar{x}) \circ S_C(\bar{x})$)

To compute the numerator of this equation, which isolates rivalrous regions, the perceptual signals ($S_C(\bar{x})$) are convolved with a spatial filter, $f(\bar{x})$–a binary mask that equals one over rivalrous disk regions and zero elsewhere:

$$f(\bar{x}) = \begin{cases} 1, & x \in \text{rivalrous disk regions (both eyes)} \\ 0, & \text{otherwise.} \end{cases}$$

To compute $f(\bar{x}) \circ S_G(\bar{x})$ take the element-wise product $f(\bar{x}) \odot S_G(\bar{x})$, *i.e.,* multiplying the raw signal by the mask at each location and then spatially pooling across both rivalrous regions and both eyes by summing the products. This pooling step thus yields the total number of pixels—red or green—contained within the rivalrous disk regions across both eyes. The filter is the same for all background conditions within an experiment, and so is the numerator (Table S1).

Divisive Normalization of Rivalrous Signals

The denominator is calculated by summing the signal for each color across the entire visual stimulus, including rivalrous and non-rivalrous regions for both eyes. The last step is to divide the numerator by the denominator for each perceptual signal (i.e., red or green) and background condition (i.e., red, green, or grey).

Model Sensitivity: Ensemble Averages and Single-Trial Dynamics

To probe parameter sensitivity, five variants were generated by perturbing each parameter individually *beyond* its empirical range while holding the other four at their default values from the main text. For each single-parameter modification we ran:

1. Ensemble average: n = 100 independent simulation trials to compute the expected dominance durations under each of the three background conditions. This corresponds to an ensemble average over stochastic realizations—akin to the ensemble means used in statistical physics—to recover the model's mean behavior in the presence of noise.

2. Single-trial examples: One 60-second time course per background to illustrate the moment-to-moment dynamics and variability.

Note on single-trial examples. The individual trial traces shown below are inherently illustrative because the model incorporates stochastic gain and intrinsic noise; any single realization may deviate substantially from both the ensemble mean and from other single trials from the same condition with identical parameters.

Before exploring the sensitivity of the model to each tunable parameter, a summary of that parameter, the relevant equation from the main text, and the default value for that parameter will be given. There are five core parameters (noise bounds [*a, b*], *g*, gain bias, τ_D, and τ_R) that govern the stochasticity and dynamics of the rivalry model. Each default value was chosen to reflect empirical estimates of neural time-constants, attentional gain, and noise levels observed in human perception and neurophysiology. Together, these parameters shape the balance between adaptation, recovery, multiplicative amplification, and stochastic fluctuations, producing the alternation dynamics examined in our simulations.

Finally, to facilitate comparison, Figure S1 shows the ensemble average and single-trial example plots for the model with the default values reported in the main text (Figure 5).

Background	Experiment	$S_R(\bar{x})$	$S_G(\bar{x})$	$\hat{S}_R$	$\hat{S}_G$
Red	1	228448	15690	$\frac{15690}{228448} \approx 0.0686$	$\frac{15690}{15690} \approx 1.0000$
Green	1	15690	228448	$\frac{15690}{15690} \approx 1.0000$	$\frac{15690}{228448} \approx 0.0686$
Grey	1	15690	15690	$\frac{15690}{15690} \approx 1.0000$	$\frac{15690}{15690} \approx 1.0000$
Rivalrous	2	228448	228448	$\frac{228448}{228448} \approx 1.0000$	$\frac{228448}{228448} \approx 1.0000$

Table S1. Raw and normalized sensory signals for each stimulus condition
The table lists, under red, green, neutral, and rivalrous (Experiment 2) background conditions, the raw pixel counts ($S_R(\bar{x})$, $S_G(\bar{x})$), which represent the total pixel count corresponding to the color signal. After applying the binary mask $f(\bar{x})$, the less common color will have a smaller pixel count but a larger normalized signal ($\hat{S}_R$, $\hat{S}_G$). Note, Experiment 1's grey background condition and Experiment 2's rivalrous background condition do not have a less common color, and both conditions produce normalized signals near 1.

Original Model: Trial-to-Trial Variability in Single Trials

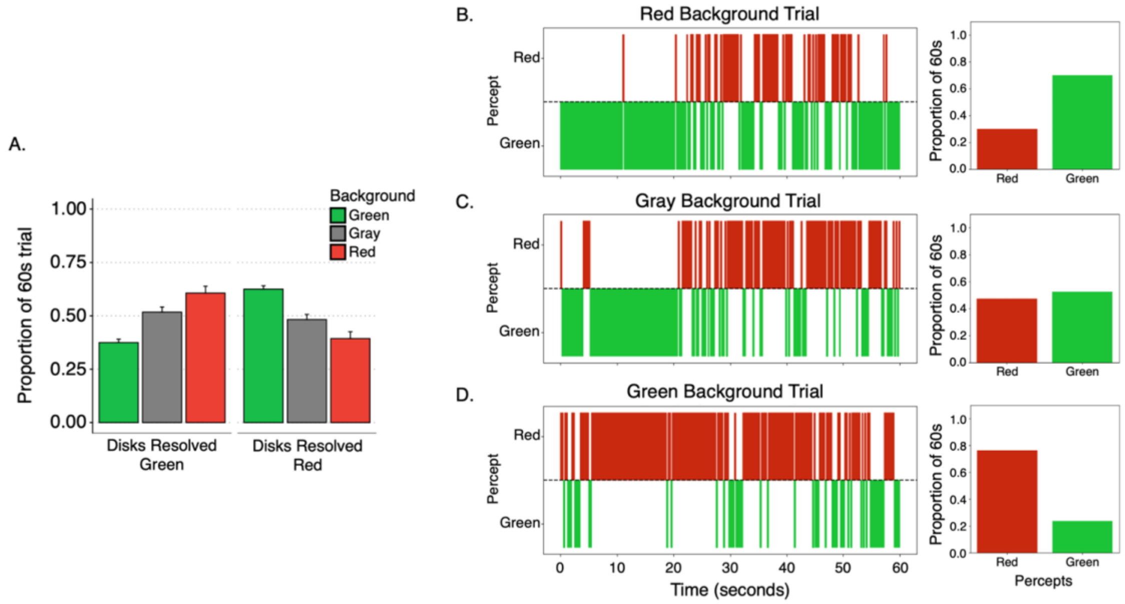

Figure S1. Ensemble and single-trial dynamics of the original model.
A. Aggregate dominance proportions for green-disk (left cluster) and red-disk (right cluster) percepts under gray (gray bars), green (green bars), and red (red bars) backgrounds, averaged over n = 100 runs. Error bars denote the standard deviation from the mean.
B–D. Single-trial (60-second) simulations for (B) red, (C) gray, and (D) green backgrounds. In each row, the left subplot shows the instantaneous percept (green = green-dominant; red = red-dominant; dashed midline is the decision boundary), and the right subplot is a bar chart showing the total proportion of time each percept dominated over the trial. These exemplars illustrate the model's stochastic gain and intrinsic noise: although the aggregate staircase pattern emerges in (A), individual trials exhibit substantial variability in both switching dynamics and net dominance.

Sensitivity to Noise Bounds

The bounds ($[a, b]$) on the truncated standard normal distribution $\mathcal{N}(0, \sigma^2)$ control the level of allowable neural noise (η_C), where the subscript on η_C indicates independent noise for each perceptual signal (i.e., S_R or S_G). Varying the bounds $[a, b]$ controls the maximum and typical noise magnitude as a fraction of the current signal. Here, a random variable (ξ) is drawn from a truncated normal distribution, which determines the contribution of noise as a proportion of the current signal. This is formalized as

$$\eta_c = S_C \cdot \xi_c, \xi \sim \mathcal{N}(0, \sigma^2)_{[a,b]}, \quad (1.3)$$

where $\mathcal{N}(0, \sigma = 0.20)_{[a = -0.35, \ b = 0.35]}$.

For the default ("moderate") noise model results, bounding parameters were set at ±0.35, meaning that while it is possible to draw a random variable that could influence the signal by ±35%, the majority (68%) of random variables drawn would fall between 0 and ±20%.

To examine the influence of the level of intrinsic neural noise on the simulated rivalry dynamics and perceptual dominance at the single-trial level and ensemble model behavior, two extreme noise levels were chosen. For each level of noise (low and high), we (1) computed mean dominance proportions over $n = 100$ runs for each background condition (grey, green, red), and (2) plotted a representative single-trial time course per background alongside a bar plot displaying a total-dominance summary for the single trial.

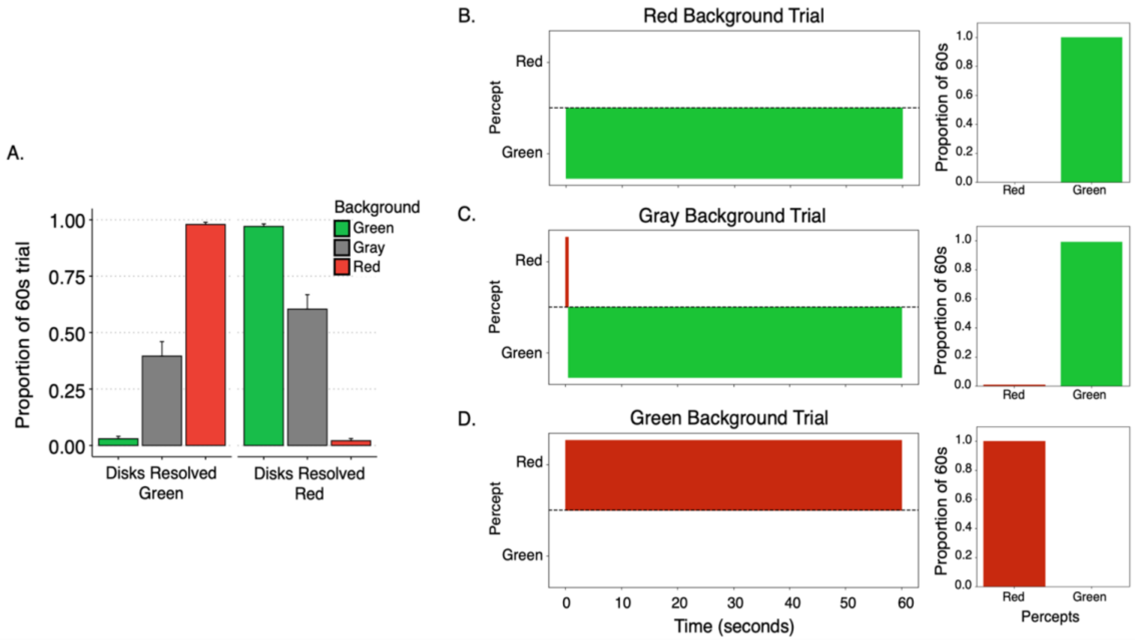

Figure S2. Low Noise Level $\mathcal{N}(0, \sigma = 0.03)$

A. Mean dominance fractions (red, gray, and green backgrounds; n = 100, error bars = standard deviation). The ensemble-average plot shows that when noise is too low, the model's normalization-driven dominance bias becomes stronger than most empirical observations.

B–D. Single trials for red (B), gray (C), and green (D) background conditions. Left subplots show the time course of the instantaneous perceptual state (green = green-dominant; red = red-dominant). The dashed horizontal midline marks the decision boundary between the two states. Right subplots show the total proportion of time red and green dominated perception. Consistent with empirical evidence and other models of rivalry dynamics, noise plays a crucial role in driving perceptual swaps. Without noise, the model becomes deterministic. To model a low noise level, bounding parameters $[a, b]$ were set to ± 0.05, yielding an effective standard deviation, σ = 0.03. Therefore, noise could occasionally reach ±5% of the signal, but about 68% of the samples drawn lie within ±3%, generating minimal perturbations.

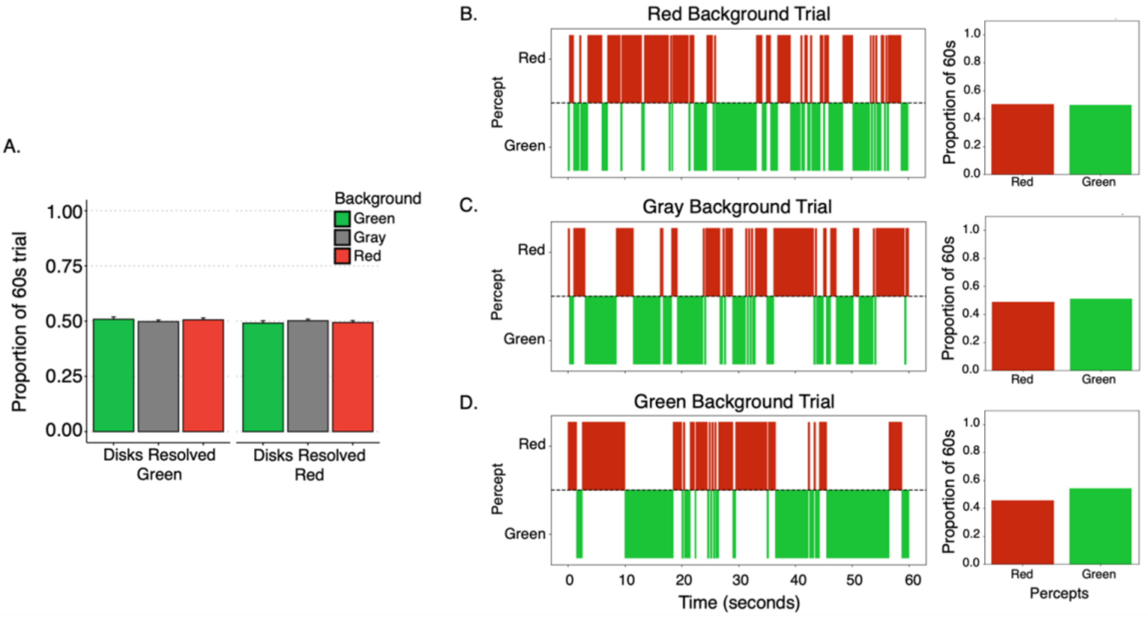

Figure S3. High Noise Level $\mathcal{N}(0, \sigma = 0.54)$

A. Mean dominance fractions (red, gray, and green backgrounds; n = 100, error bars = standard deviation). The ensemble-average plot shows that when noise is too high, the model is swamped by noise and the bias in perceptual dominance is diminished.

B–D. Single trials for red (B), gray (C), and green (D) background conditions. Left subplots show the time course of the instantaneous perceptual state (green = green-dominant; red = red-dominant). The dashed horizontal midline marks the decision boundary between the two states. Right subplots show the total proportion of time red and green dominated perception. To model a high noise level, bounding parameters $[a, b]$ were set to ± 1.00, yielding an effective standard deviation (σ) of 0.54, such that noise could reach ±100% of the signal but that 68% of the samples drawn would lie within ±54%. This noise level generated larger perturbations than the default settings. This additional stochasticity overwhelmed the deterministic part of the model, the stimulus-dependent normalization, as well as any perceptual stability provided by attentional gain and gain bias.

Multiplicative Gain Amplification

In the model, the gain parameter, g, multiplicatively amplifies a selected channel's normalized signal (Eq. 2.1). The selected signal is not necessarily the current dominant percept. Gain is randomly applied to one of the two channels, with a slight bias towards the dominant (see the description of the gain bias parameter).

$$P_D \text{ or } P_S = g(P_D \text{ or } P_S) \tag{2.1}$$

In the original simulations, gain (g) was set to 1.3, yielding a 30% amplification of the selected signal. To assess how gain magnitude shapes both ensemble averages and single-trial dynamics, we compared a low gain of $g = 1.05$ (5% amplification) to a high gain of $g = 1.95$ (95% amplification). For each gain setting, the mean dominance proportions over $n = 100$ independent runs under each background condition were computed. Then, one representative 60-second trial per background was plotted, with its total dominance fractions summarized in an adjacent bar chart.

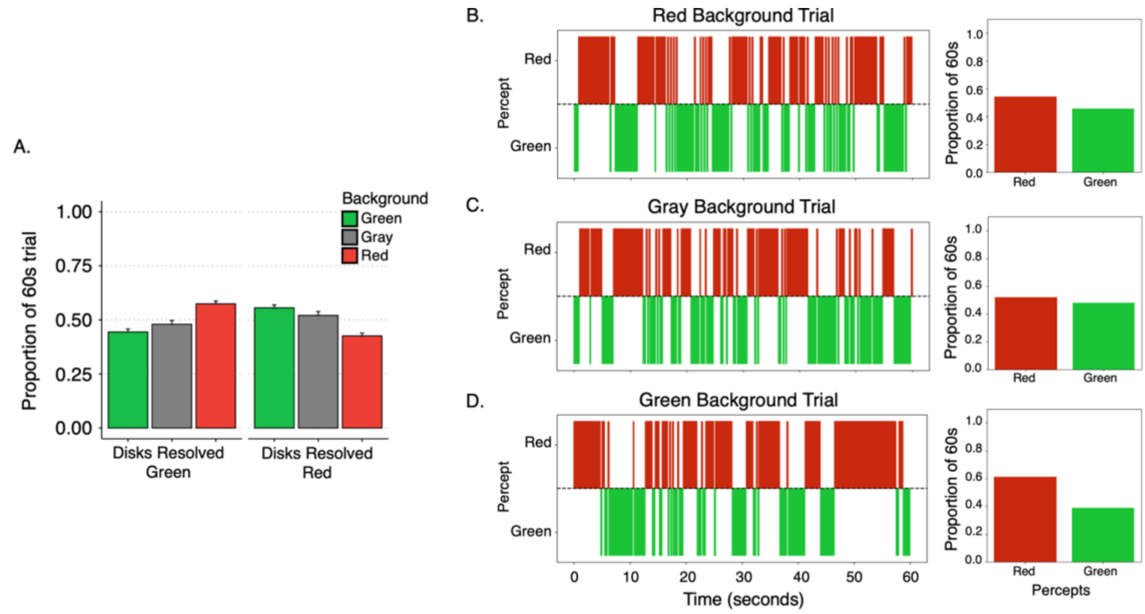

Figure S4. Minimal Attentional Gain

A. Mean dominance fractions (red, gray, and green backgrounds; n = 100, error bars = standard deviation). With g reduced to 1.05, the staircase-like modulation by background context is markedly attenuated compared to the original model.

B–D. Representative 60-second single-trial dynamics for red (B), gray (C), and green (D) backgrounds. Left panels plot the instantaneous perceptual state (green = green-dominant; red = red-dominant; dashed line = decision boundary), and right panels show the total dominance fraction of each percept. Low gain diminishes the stabilizing effect of biased attentional amplification, producing more balanced perceptual distributions and greater variability and more perceptual switching across individual runs.

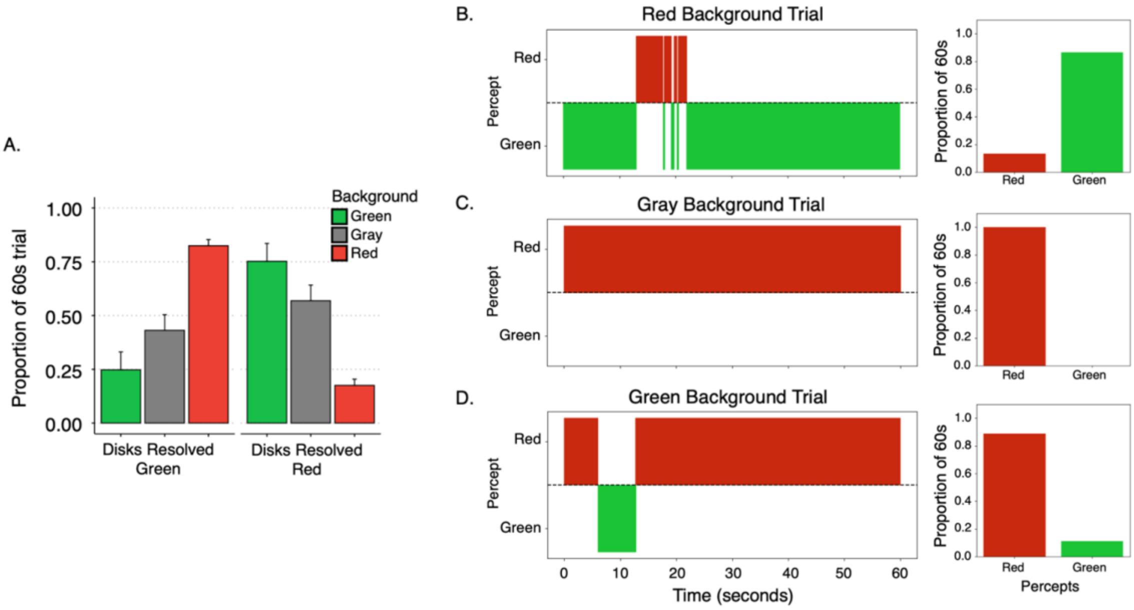

Figure S5. High Attentional Gain

A. Mean dominance proportions for green-disk (left cluster) and red-disk (right cluster) percepts under green, gray, and red backgrounds, averaged over n = 100 simulations (error bars = ±1 SD). With g elevated to 1.95, the staircase modulation by background is dramatically amplified: the background-contrasted disks dominate over 75% of the time, while the background-matched disk is suppressed.

B–D. Representative 60-second single-trial dynamics for red (B), gray (C), and green (D) backgrounds. Left panels plot the instantaneous perceptual state (green = green-dominant; red = red-dominant; dashed line = decision boundary), and right panels show the total proportion of time in each percept. Under high gain, biased amplification overwhelms adaptation and noise, resulting in near-deterministic dominance of the background-contrasted percept and minimal perceptual switching.

Gain Bias

In the model, gain bias controls which channel receives multiplicative amplification at each time step. In the original simulations, the dominant percept was chosen with 55% probability (a 5% bias above chance) and the suppressed percept with 45%.

To assess how different bias strengths shape ensemble averages and single-trial dynamics, we compared three regimes: the default moderate bias [0.55, 0.45], an unbiased control [0.50, 0.50], and an extreme bias [0.90, 0.10] in which the dominant percept is selected 90 % of the time. For each bias condition, total dominance proportions were first averaged over n = 100 runs under each background (grey, green, red). Then, one representative 60-second trial per background was plotted, with its overall dominance fractions summarized in an adjacent bar chart.

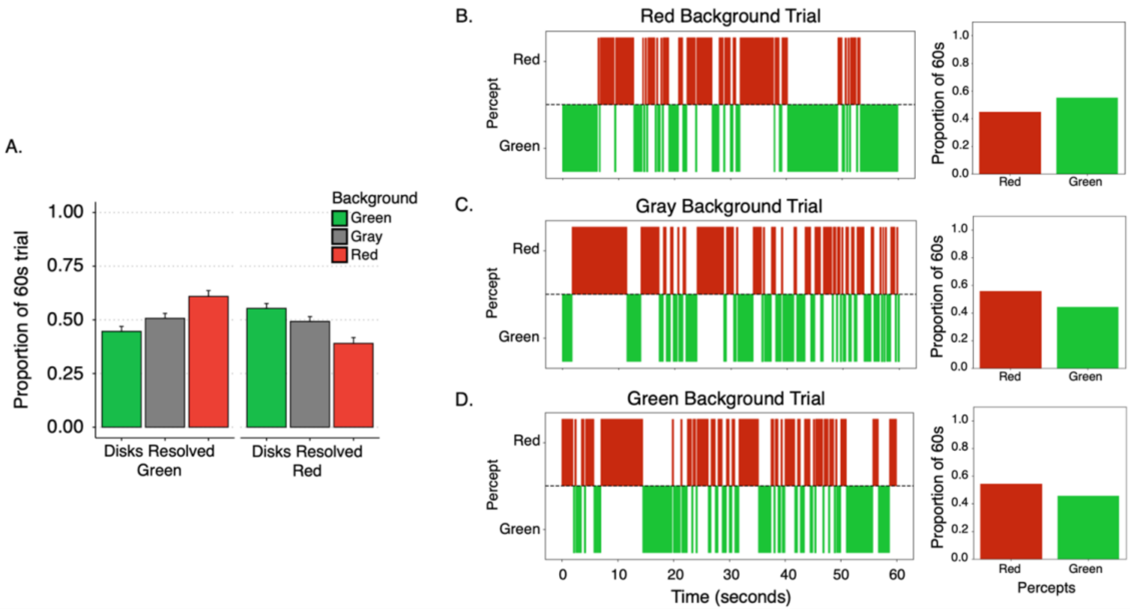

Figure S6. No Gain Bias

A. Mean dominance proportions for green-disk (left cluster) and red-disk (right cluster) percepts under green, gray, and red backgrounds, averaged over n = 100 runs (error bars = ±1 SD). With gain applied at random, the staircase-like modulation by background is markedly reduced, and the ensemble pattern closely resembles the minimum gain amplification model (Figure S4), since unbiased amplification yields no net advantage.

B-D. Representative 60-second single-trial dynamics for red (B), gray (C), and green (D) backgrounds. Left panels show instantaneous perceptual state (green = green-dominant; red = red-dominant; dashed line = decision boundary); right panels report total dominance fractions. As with minimal gain amplification, the lack of gain bias enables noise to have a larger influence, inducing a higher frequency of perceptual swaps.

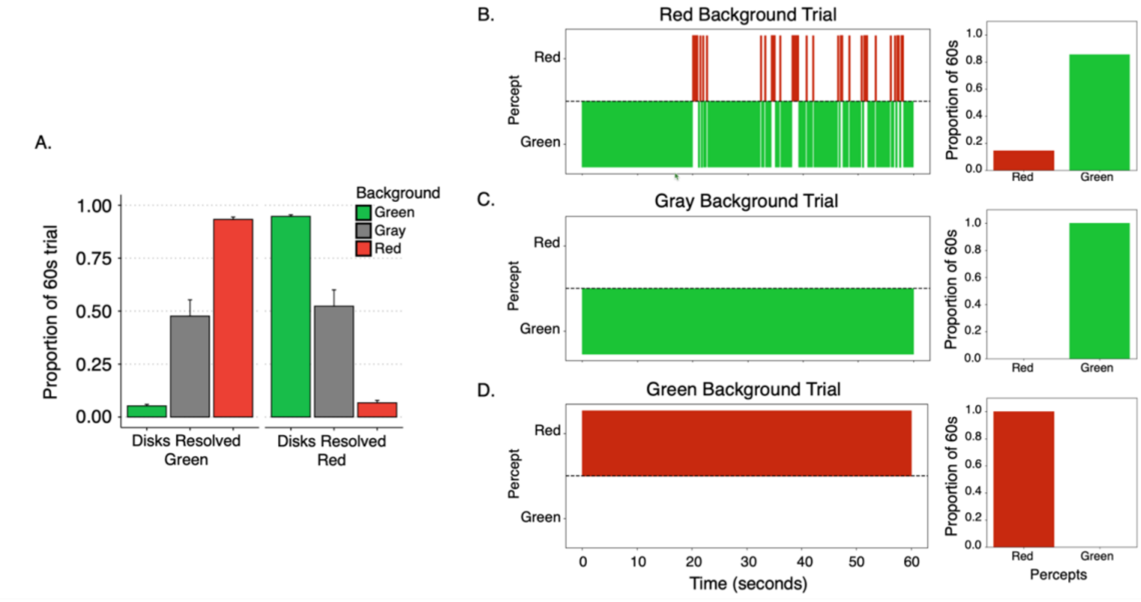

Figure S7. Significant Gain Bias

A. Mean dominance proportions for green-disc and red-disc percepts under green, gray, and red backgrounds, averaged over n = 100 runs (error bars = ±1 SD). With a 90% bias toward the dominant percept, the staircase modulation is exaggerated, yielding nearly deterministic ensemble results that parallel those of the high-gain amplification model (Figure S5).

B-D. Representative 60-second single-trial dynamics for red (B), gray (C), and green (D) backgrounds. Left panels show instantaneous perceptual state (green = green-dominant; red = red-dominant; dashed line = decision boundary); right panels report total dominance fractions. Extreme bias drives near-deterministic dominance of the background-contrasted percept. As with the single-trial results of the high-gain amplification model, biased amplification overwhelms the stochasticity provided by adaptation and noise.

Adaptation-constant (τ_D)

The adaptation constant (τ_D) governs the exponential decay of the dominant representation's (P_D) signal strength over time. The default value of the decay rate constant was set to 2.5 seconds. Adaptation is expressed in its differential form to emphasize it as a recurrent process with time-varying stochasticity:

$$\frac{dP_D(t)}{dt} = \frac{-P_D(t)}{\tau_D} + \eta_D(t). \tag{3.1}$$

To investigate the impact of the adaptation rate constant on simulated rivalry dynamics at the single-trial level and ensemble model behavior, two extreme values for the decay constant (τ_D) were tested: an unnaturally fast τ_D of 0.1 ms and a very slow τ_D of 20 minutes. For each setting, (1) total dominance proportions were averaged over $n = 100$ runs for each of the three background conditions, and (2) to demonstrate model dynamics, a single trial from each background condition was plotted over time, with the total proportions summarized in an adjacent plot.

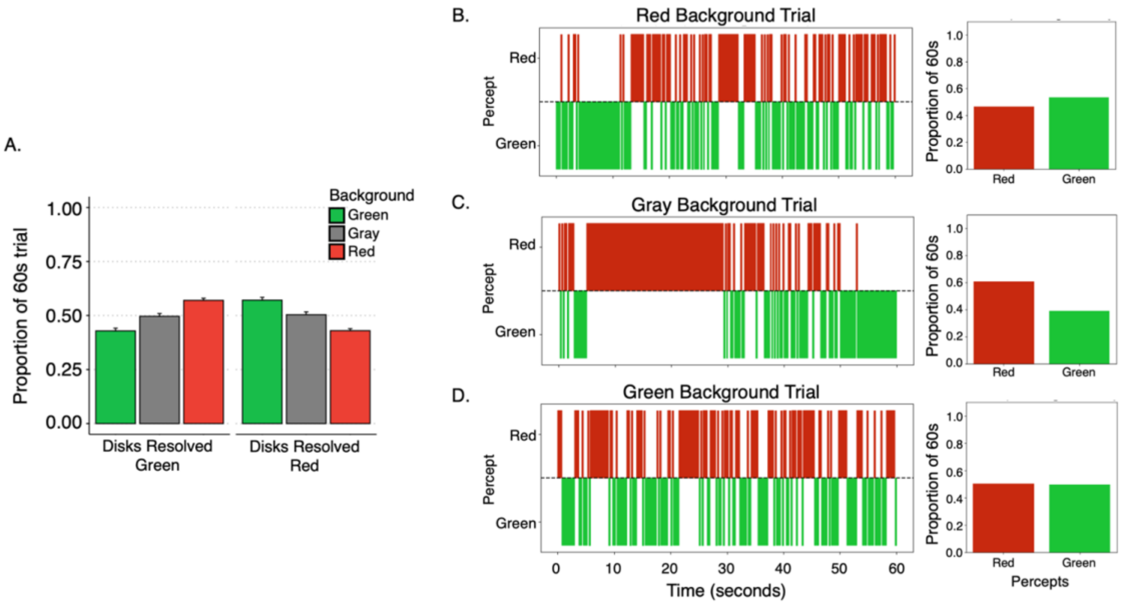

Figure S8. Fast adaptation constant (τ_D = 0.1 ms).

A. Mean dominance fractions (red, gray, and green backgrounds; *n* = 100, error bars = standard deviation). With τ_D reduced from 2.5 s to 0.1 ms, adaptation is effectively instantaneous: the staircase modulation by background context is attenuated due to rapid perceptual switching.

B–D. Single trials for red (B), gray (C), and green (D) background conditions. Left subplots show the time course of the instantaneous perceptual state (green = green-dominant; red = red-dominant). The dashed horizontal midline marks the decision boundary between the two states. Right subplots show the total proportion of time red and green dominated perception. Fast adaptation often produced rapid perceptual switching and inconsistent trial outcomes. The dominant behavior of the model is severely dampened by having instantaneous adaptation.

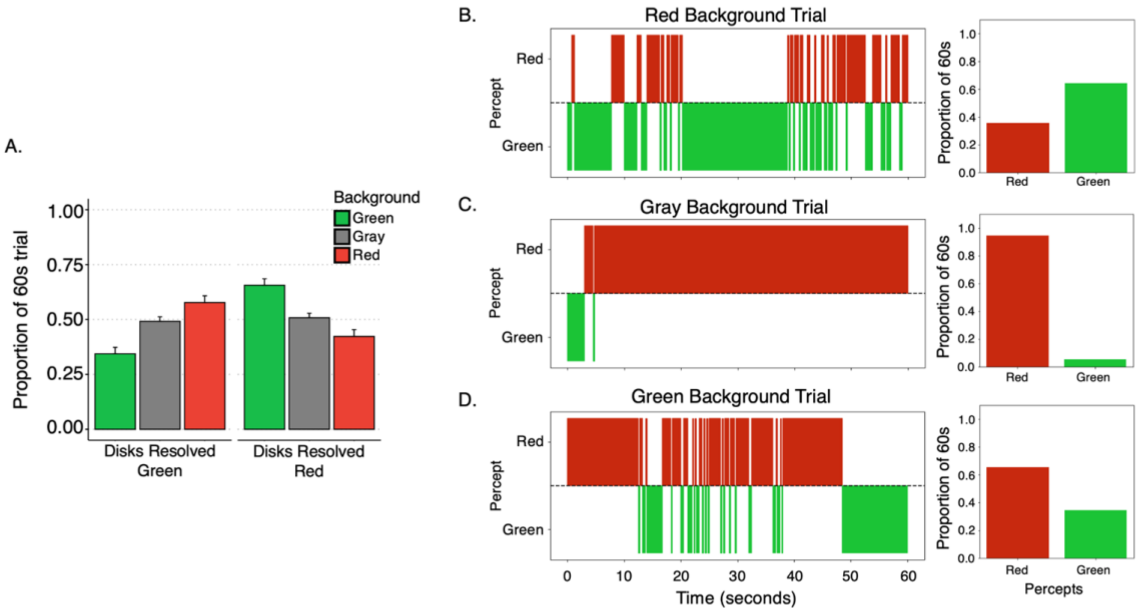

Figure S9. Slow adaptation constant (τ_D = 20 minutes).

A. Mean dominance fractions (red, gray, and green backgrounds; $n = 100$, error bars = standard deviation). With τ_D increased from 2.5 s to 20 minutes, the rate of adaptation is outside the normal range for the short-term adaptation possible with the experimental stimulus used. The ensemble results are very similar to the original model. With such a slow decay, adaptation doesn't affect the simulation results, and the dominant model behavior determined by the normalization term of the model drives the results.

B–D. Single trials for red (B), gray (C), and green (D) background conditions. Left subplots show the time course of the instantaneous perceptual state (green = green-dominant; red = red-dominant). The dashed horizontal midline marks the decision boundary between the two states. Right subplots show the total proportion of time red and green dominated perception. Slower adaptation resulted in slightly longer dominance durations for the red and green backgrounds compared to the original model. The results were often deterministic at the single-trial level for neutral background trials, which lack a strong drive from the normalization step, with one color dominating for all or most of the trial (C).

Recovery Adaptation Constant (τ_R)

Recovery constant (τ_R) governs the exponential recovery of the suppressed representation's (P_R) signal strength over time. It is likewise represented in a differential form to emphasize its recurrency and time-varying stochasticity:

$$\frac{dP_S(t)}{dt} = \frac{P_M - P_S(t)}{\tau_R} + \eta_S(t). \tag{3.2}$$

The default value of τ_R in the original model was set to 1.5 seconds. To explore the influence of the recovery rate constant on the simulated rivalry dynamics at the single-trial level and ensemble model behavior, two extreme values for the decay constant (τ_R) were tested: an unnaturally fast τ_R of 0.1 ms and a very slow τ_R of 20 minutes. For each setting, (1) total dominance proportions were averaged over n = 100 runs for each of the three background conditions, and (2) to demonstrate model dynamics, a single trial from each background condition was plotted over time, with the total proportions summarized in an adjacent plot.

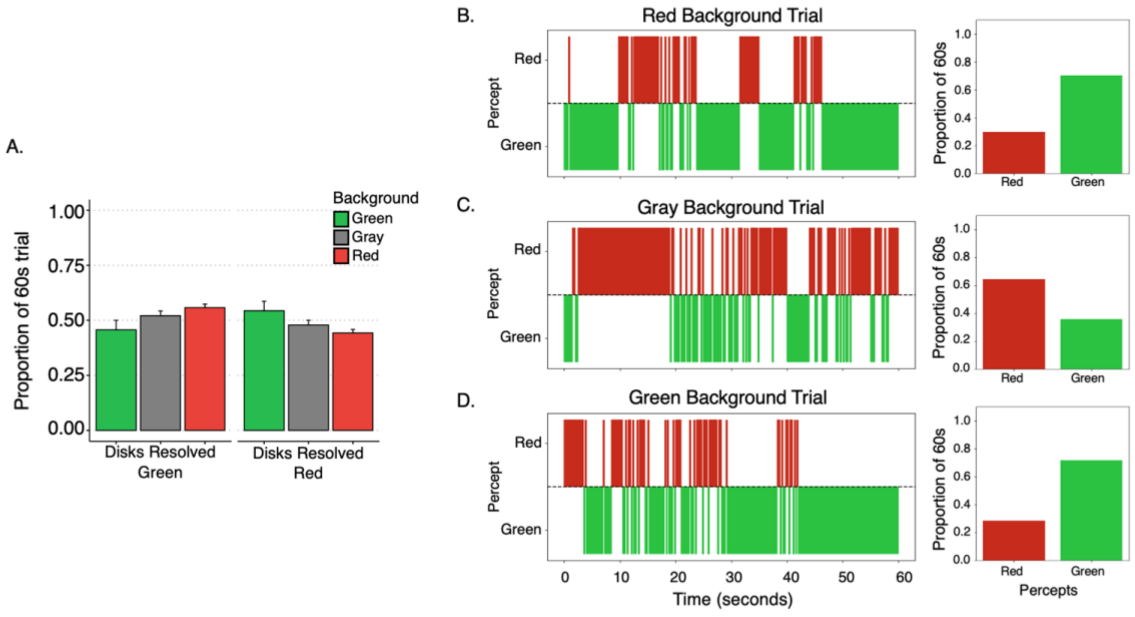

Figure S10. Fast recovery constant (τ_R = 0.1 ms).

A. Mean dominance fractions (red, gray, and green backgrounds; *n* = 100, error bars = standard deviation). When τ_R is shortened from 1.5 s to 0.1 ms, adaptation is reset virtually instantaneously, preventing any cumulative attenuation of the dominant signal. As a result, the characteristic staircase modulation by background context is heavily attenuated due to rapid perceptual switching and increased variance across single-trial runs.

B–D. Single trials for red (B), gray (C), and green (D) background conditions. Left subplots show the time course of the instantaneous perceptual state (green = green-dominant; red = red-dominant). The dashed horizontal midline marks the decision boundary between the two states. Right subplots show the total proportion of time red and green dominated perception. Instant recovery eliminates the braking effect of adaptation, so gain-driven fluctuations dominate—leading to rapid, erratic alternations punctuated by sporadic long dominance durations and markedly higher single-trial variance.

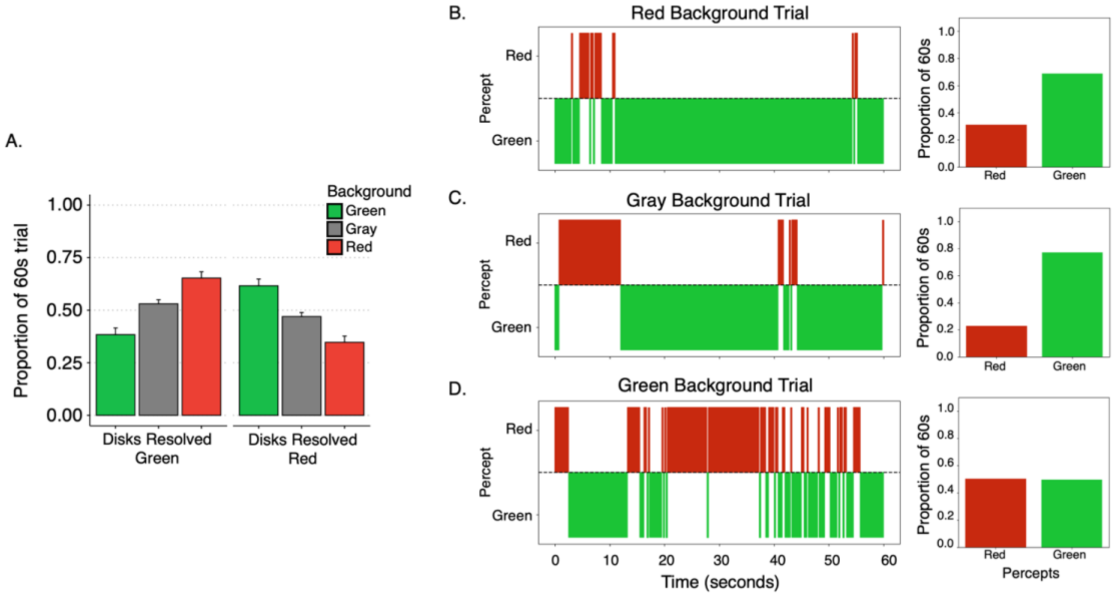

Figure S11. Slow recovery constant (τ_R = 20 minutes)

A. Mean dominance fractions (red, gray, and green backgrounds; *n* = 100, error bars = standard deviation). Here, τ_R is lengthened from 1.5 s to 20 min—far beyond the timescale of a 60 s trial—so that once a channel adapts, it effectively never recovers within each run. The adapted signals decay together throughout the trial, and the overall balance of dominance remains similar to the original model's ensemble pattern, despite the extreme recovery setting.

B–D. Single trials for red (B), gray (C), and green (D) background conditions. Left subplots show the time course of the instantaneous perceptual state (green = green-dominant; red = red-dominant). The dashed horizontal midline marks the decision boundary between the two states. Right subplots show the total proportion of time red and green dominated perception. Slower recovery dampened perceptual switching, and the results were often nearly deterministic at the single-trial level for neutral background trials. Since signals adapt but do not functionally recover, the influences of biased gain amplification and stochastic [illegible]
